# A conditional mutation in a wheat (*Triticum aestivum* L.) gene regulating root morphology

**DOI:** 10.1007/s00122-024-04555-7

**Published:** 2024-02-12

**Authors:** Deying Zeng, Brett Ford, Jaroslav Doležel, Miroslava Karafiátová, Mathew J. Hayden, Tina M. Rathjen, Timothy S. George, Lawrie K. Brown, Peter R. Ryan, Filomena A. Pettolino, Ulrike Mathesius, Emmanuel Delhaize

**Affiliations:** 1 Department of Biological Science, College of Life Sciences, Sichuan Normal University, Chengdu, Sichuan 610101 China; 2 Present Address: Grains Research and Development Corporation, Barton, ACT 2600 Australia; 3 Centre of Plant Structural and Functional Genomics, Institute of Experimental Botany of the Czech Academy of Sciences, Olomouc, Czech Republic; 4 Department of Jobs, Precincts and Regions, Agriculture Victoria Research, AgriBio, Bundoora, VIC Australia; 5 School of Applied Systems Biology, La Trobe University, Bundoora, VIC Australia; 6 CSIRO Agriculture & Food, PO Box 1700, Canberra, ACT 2601 Australia; 7 James Hutton Institute, Invergowrie, Dundee, DD2 5DA UK; 8 Research School of Biology, The Australian National University, Canberra, ACT 2601 Australia

## Abstract

**Key message:**

Characterisation and genetic mapping of a key gene defining root morphology in bread wheat.

**Abstract:**

Root morphology is central to plants for the efficient uptake up of soil water and mineral nutrients. Here we describe a conditional mutant of hexaploid wheat (*Triticum aestivum* L.) that when grown in soil with high Ca^2+^ develops a larger rhizosheath accompanied with shorter roots than the wild type. In wheat, rhizosheath size is a reliable surrogate for root hair length and this was verified in the mutant which possessed longer root hairs than the wild type when grown in high Ca^2+^ soil. We named the mutant *Stumpy* and showed it to be due to a single semi-dominant mutation. The short root phenotype at high Ca^2+^ was due to reduced cellular elongation which might also explain the long root hair phenotype. Analysis of root cell walls showed that the polysaccharide composition of *Stumpy* roots is remodelled when grown at non-permissive (high) Ca^2+^ concentrations. The mutation mapped to chromosome 7B and sequencing of the 7B chromosomes in both wild type and *Stumpy* identified a candidate gene underlying the *Stumpy* mutation. As part of the process to determine whether the candidate gene was causative, we identified wheat lines in a Cadenza TILLING population with large rhizosheaths but accompanied with normal root length. This finding illustrates the potential of manipulating the gene to disconnect root length from root hair length as a means of developing wheat lines with improved efficiency of nutrient and water uptake. The *Stumpy* mutant will be valuable for understanding the mechanisms that regulate root morphology in wheat.

**Supplementary Information:**

The online version contains supplementary material available at 10.1007/s00122-024-04555-7.

## Introduction

Roots are the primary point of contact between plants and the soil in which they reside and are critical for acquiring water and mineral nutrients. Root morphology is key in how efficiently plants acquire these resources, especially when they become limiting. For example, plants with long root hairs are better able than those with short root hairs to take up a poorly-mobile nutrient from soil such as phosphate (Gahoonia et al. 1997; Gahoonia and Nielsen 2003; Krasilnikoff et al. 2003; Zhang et al. 2018).

Hexaploid wheat (*Triticum aestivum* L.) is one of mankind’s major cultivated crops and provides a large proportion of the carbohydrates consumed by humans (Pena-Bautista et al. 2017). Much of the research aimed at improving wheat germplasm has focussed on shoots. For instance, the *Rht* dwarfing genes proved to be instrumental in contributing to the dramatic increases in grain yields of the Green Revolution (Hedden 2003). Genes underlying other shoot traits such as vernalisation, disease resistance and grain quality have also been identified (Li et al. 2020). By contrast, there are few examples of major genes affecting roots that have been implemented in wheat improvement. Notable examples are genes that provide protection from soil toxins that occur under specific conditions such as boron toxicity (Schnurbusch et al. 2007) and aluminium on acid soils (Delhaize et al. 2012).

In contrast to a number of diploid species, there are few examples of root mutants in hexaploid wheat and this can be attributed to (a) the difficulty of working on roots compared to shoots and (b) a reluctance of researchers to use a species with a large and polyploid genome as a model. The release of an updated and annotated version of the genome sequence along with transcriptomics and improved transformation methods goes some way towards alleviating the difficulty of using hexaploid wheat as a model organism (Adamski et al. 2020; International Wheat Genome Sequencing Consortium 2018). In addition, the availability of heavily mutagenised targeting induced local lesions in genomes (TILLING) populations can enable recessive mutations to be combined for each of the genomes to unmask what has been described as “hidden variation” (Krasileva et al. 2017). Nevertheless, with a polyploid genome of size about 16 Gb the wheat genome still represents a considerable challenge for cloning genes based solely on a mutant phenotype (Uauy 2017).

Here we describe the identification of a single-gene root mutant of wheat. The mutant phenotype is conditional on the Ca^2+^ concentration of the growing medium and, under non-permissive Ca^2+^ concentrations, has longer root hairs and shorter roots than the wild type (WT). We have identified a candidate for the mutated gene which is predicted to encode a large transmembrane protein. The gene underlying the mutation appears to be key in controlling the morphology of wheat roots by regulating cell elongation.

## Materials and methods

### Screening of mutagenised populations

Grain of the bread wheat cultivar (cv) Westonia was treated with 1 mM sodium azide using a previously described method (Chandler and Harding 2013). Up to twenty M_3_ families were kept separated from one another to ensure that mutants identified from different families could be maintained as independently derived mutants. A WT line of cv Westonia was developed by single seed descent to ensure a consistent genetic background and used for backcrossing and generation of a mapping population. M_3_ families were screened using a modified rhizosheath assay. The rhizosheath is defined as the soil that remains adhered to roots and in wheat is correlated with root hair length on seedlings grown in soil (Delhaize et al. 2015). Three to four pre-germinated grains were sown in small pots that contained soil amended with lime (30 g CaCO_3_/ kg soil) as described previously (Delhaize et al. 2015). After 3 days of growth, seedlings were tipped out into a tray and inspected visually for rhizosheath size. Seedlings with larger rhizosheaths than WT were transferred to pots of compost mix and grown to maturity. To increase throughput using visual and quantified screens, seedlings were grown in trays instead of small pots which enabled 50 seedlings to be screened per tray. Grain was collected from seedlings selected as having large rhizosheaths and progeny assayed for rhizosheath size.

Sequence from the Cadenza TILLING population described by Krasileva et al. (2017) was used in a BLAST (https://github.com/homonecloco/bioruby-wheat-db) to identify lines that had mutations in *TraesCS7B03G0323100* (IWGSC RefSeq v2.1annotation, http://wheat-urgi.versailles.inra.fr/Seq-Repository/Assemblies). Seed samples of selected lines were obtained through SeedStor (https://www.seedstor.ac.uk). For experiments undertaken in Canberra Australia, a preliminary visual screen of rhizosheath size was undertaken on limed soil under quarantine conditions and seedlings with large rhizosheaths selected for bulking of seed. The bulked seed from separate plants was then screened for rhizosheath size to confirm the large rhizosheaths. For experiments undertaken in Dundee Scotland, also on limed soil, the seed obtained from SeedStor was used directly in rhizosheath assays to quantify rhizosheath size. *TraesCS7B03G0323100* was sequenced in a selection of Cadenza TILLING lines to confirm the presence of mutations.

### Mapping

The *Stumpy* mutant was crossed to WT lines that had been produced by single seed descent. *Stumpy* used in physiological experiments had been backcrossed four times to WT cv Westonia except where specified in figure legends. To determine the genetics and to map the trait, *Stumpy* was crossed to WT in cv Westonia and cv Chara backgrounds. To map the genetic location of the trait, seedlings of the *Stumpy* by cv Chara cross were selected in the F_2_ that had either WT or *Stumpy* phenotypes. The phenotypes of selected seedlings were verified in the F_3_ generation and 18 lines homozygous for WT or *Stumpy* were combined into two groups and subjected to bulked segregant analysis (Michelmore et al. 1991) using a 90 K single nucleotide polymorphism (SNP) chip. A pair of contrasting bulks was prepared by pooling equal amounts of genomic DNA from F_3_ lines that were selected based on phenotype and verified to be homozygous for *Stumpy* or WT. An artificial F_1_ sample was prepared by combining an equal amount of DNA from each of the parental lines. The bulked DNA samples, artificial F_1_, cv Chara and *Stumpy* (cv Westonia background) were genotyped for 90,000 gene-based SNPs using the Infinium iSelect 90 K wheat bead chip array (Wang et al. 2014), following the manufacturer’s instructions (Illumina Ltd). The SNPs were assessed for putative linkage by comparing the normalised theta values for each sample as described by previously (Hyten et al. 2008). SNPs were considered to be putatively linked to the mutation when the normalised theta values for the *Stumpy* bulk and *Stumpy* (cv Westonia background, control line), and WT bulk and cv Chara were similar, and the normalised theta value for the artificial F_1_ samples was about halfway between that of the other samples. Putatively linked markers were confirmed by manual inspection using GenomeStudio v2011.1 software (Illumina Ltd). SNPs between cv Westonia and cv Chara within this region were used in Kompetitive allele specific PCR (KASP) assays to verify co-segregation with the *Stumpy* trait and to map the gene. The *Stumpy* trait initially appeared to be dominant, so seedlings in the F_2_ of the Stumpy by cv Chara cross that had a WT phenotype were selected and SNPs identified as being polymorphic between parents were used in KASP assays to map the mutation. The genotypes of selected seedlings in the F_2_ were verified in the F_3_ generation.

To identify candidate genes, chromosome 7B was purified using flow cytometry from the parental cv Westiona and a *Stumpy* line that had been backcrossed four times to the parental line. Chromosome 7B was purified and sequenced from each line using previously described methods (Sánchez-Martín et al. 2016). The sequences of both genotypes of chromosome 7B between the two closest flanking markers to the *Stumpy* mutation were compared to identify SNPs consistent with the action of Na azide as a mutagen. DNA prepared from isolated chromosomes was sequenced, and raw Illumina reads from chromosome 7B DNA of cv Westiona and *Stumpy* were quality trimmed using Trimmomatic v0.32 with the default settings for paired end reads (Bolger et al. 2014). Quality trimmed reads were mapped to the Chinese Spring RefSeq v1.0 assembly (International Wheat Genome Sequencing Consortium 2018) using Bowtie2 v2.2.9 with settings set to very-sensitive-local (Langmead and Salzberg 2012). SAM files were converted to BAM format using Samtools (Li et al. 2009). KASP markers identified from the mapping to flank the region of chromosome 7B containing the *Stumpy* mutation were anchored to the Chinese Spring RefSeqv1.0 assembly. SNPs in this region were identified by visually inspecting the mapped sequences of Westonia and *Stumpy* in JBrowse (https://urgi.versailles.inra.fr/jbrowseiwgsc/). C to T and G to A transitions were considered as putative casual mutation of the *Stumpy* phenotype consistent with the action of Na azide as a mutagen. Subsequent to the release of Chinese Spring RefSeq v2.1 assembly, the location of the *Stumpy* mutation was located on the updated genome using the closest flanking markers.

### Hydroponics

Seedlings were grown in hydroponic culture to determine the effect that mineral nutrients had on the *Stumpy* phenotype. Black plastic containers (31 cm × 24 cm × 32 cm) that took 18 L of nutrient solution maintained at pH 6 was as described previously (Delhaize et al. 2004) except that 0.5 mM NH_4_(NO_3_) was substituted with NH_4_Cl and KNO_3_ each at 0.5 mM. For some experiments the nutrient solution was supplemented with various concentrations of salts as indicated in figure legends. After verifying no effect on phenotype, 2-(N-morpholino)ethanesulfonic acid hemi-sodium buffer (MES) was included in the solution at 1 mM to stabilise the pH at 6.0 of experiments as specified in legends to figures. MES buffer was not included in an experiment that compared genotypes grown in nutrient solutions at a range of pH values (5, 7 and 9) that contained either 0.5 mM or 10 mM CaCl_2_.

### RNA extraction and qRT-PCR

Root tissues from WT and *Stumpy* mutant grown for 20 days in hydroponic solution with CaCl_2_ added to 0.5 mM or 10 mM were collected in liquid nitrogen. Total RNA was extracted from roots using an OminiPlant RNA Kit (CWBIO, Jiangsu, China) according to the manufacturer’s instructions, and first-strand cDNA was synthesised using the EasyScript® All-in-One First-Strand cDNA Synthesis SuperMix Kit (TransGen Biotech, Beijing, China). The cDNA was diluted with water in a 1:5 ratio, and 2 μl of diluted cDNA was used for qRT-PCR with the 2xSuperFast Universal SYBR Master Mix (CWBIO) using a CFX96 Real-Time PCR Detection System (Bio-Rad Laboratories, Inc.). The wheat *β*-*ACTIN* gene was used as an endogenous control (Paolacci et al. 2009). Primers used for qRT-PCR are listed in Table S1.

### Microscopy

Root segments were analysed with a Leica SP8 confocal laser‐scanning microscope (Leica Microsystems, Australia) equipped with a 20 × (Numeric aperture = 0.5) water immersion objective and the Leica Application Suite V3.5.

### Monosaccharide linkage analysis

Seedlings were grown in hydroponics with MES to buffer the solutions as described above and roots collected after 16 days of growth. Roots were subjected to monosaccharide linkage analysis using a previously described method (Pettolino et al. 2012). Briefly, roots were rinsed with de-ionised water, blotted dry and then ground in liquid nitrogen before storage at − 80 °C. For analysis, cell walls were prepared as alcohol insoluble residue by extracting ground tissue four times with 70% ethanol, once with chloroform, once with methanol and once with acetone before being air dried. Samples had minimal amounts of starch which enabled them to be treated without a starch-reduction step. About 2 mg of sample was carboxyl-reduced to detect uronic acids and methylesterified uronic acids while about 100 mg of sample was methylated, hydrolysed, reduced and acetylated then analysed by gas chromatography–mass spectrometry to detect partially-methylated alditol acetates.

### Auxin quantification

Roots were frozen in liquid nitrogen and ground to a fine powder with mortar and pestle. [^2^H_5_]IAA (Cambridge Isotopes Laboratory, MA, USA) was used as an internal standard in each individual sample. Auxins were extracted with 500 µl extraction solvent (methanol/propanol/glacial acetic acid, 20:79:1, v/v/v) in a sonicator bath for 30 min at 4 °C. Samples were then centrifuged at 16,100 g for 15 min. The supernatant was transferred to a fresh tube and subsequently dried in a Speedvac centrifuge. Vacuum-dried samples were resuspended with 100% methanol, vortexed for five seconds, and filtered through a Nanosep MF GHP 0.45 µm filter (Pall Life Sciences) by centrifugation. After drying, each sample was resuspended in 50 µl methanol and water (60:40, v/v). Auxins were then separated and identified by LC–MS using a Thermo UPLC Q Exactive Plus Orbitrap LC–MS/MS system (Thermo Fisher Scientific, Waltham, MA, USA) as described in Demina et al. (2019). Auxins were identified and quantified against authentic standards of indole-3-acetic acid [IAA], IAA-Alanine [IAA-Ala], IAA-Aspartate [IAA-Asp], IAA-Leucine [IAA-Leu]/IAA-Isoleucine [IAA-Ile], IAA-Phenylalanine [IAA-Phe], 4-cloro-IAA [4-Cl-IAA], IAA-Tryptophan [IAA-Trp], IAA-Valine [IAA-Val], indole-3-butyric acid [IBA] and phenylacetic acid [PAA]. Auxins were sourced as follows: IAA-Phe, IAA-Leu, IAA-Val, IAA-Trp, 4-Cl-IAA from Olchemim Ltd, Olomouc, Czech Republic; IAA-Asp, IAA-Ala, IAA-Ile, IAA, IBA, PAA from Sigma, St. Louis, MO, USA.

## Results

### Stumpy is a conditional single-gene mutant whose phenotype is controlled by the Ca^2+^ concentration of the growth medium

**Fig. 1:**
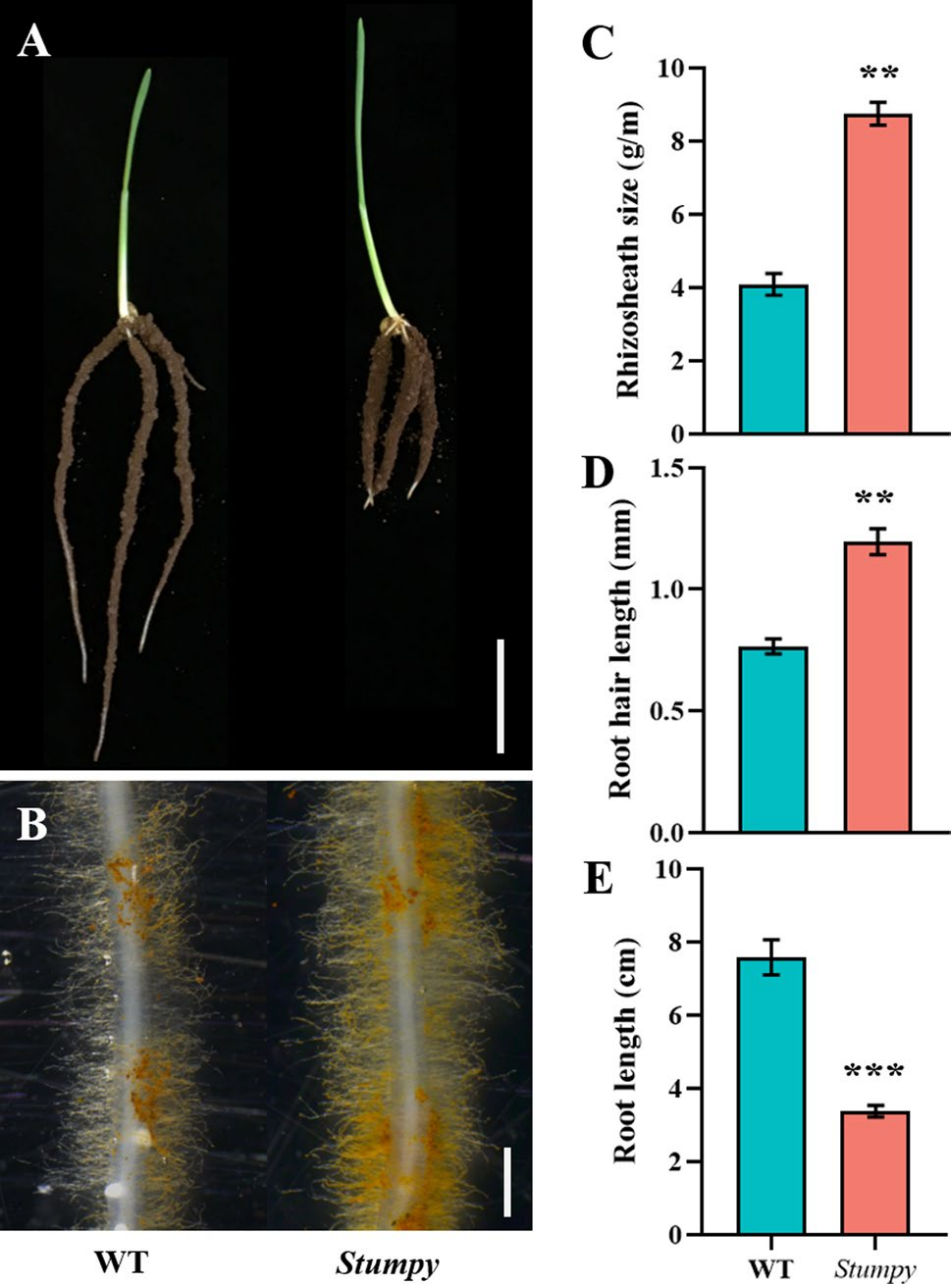
The *Stumpy* mutant has long root hairs that are associated with short roots when grown in a soil amended with lime (CaCO_3_). **A** Root phenotypes of 3-day-old WT and *Stumpy* (bar indicates 2 cm). **B** Root hair phenotypes of WT and *Stumpy* (bar indicate 1 mm). **C** Rhizosheath sizes of WT and *Stumpy*. **D** Root hair lengths of WT and *Stumpy*. **E)** Root lengths of WT and *Stumpy*. For panels **C** to **E** error bars indicate SE (*n* = 6). The *Stumpy* line was backcrossed four times to WT and *P* values for significant differences were calculated by Student’s *t* test (** *P* < 0.01; *** *P* < 0.001)

**Fig. 2:**
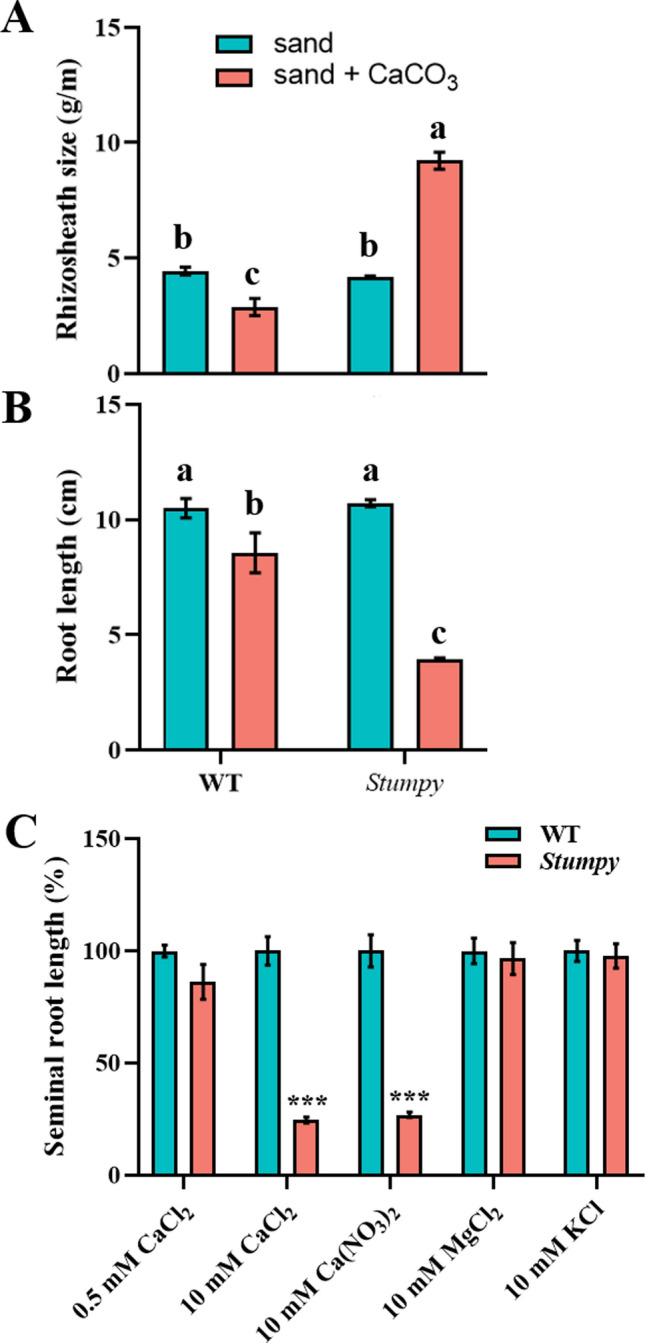
*Stumpy* is a conditional mutant with the phenotype dependant on the Ca^2+^ concentration of the growing medium. **A** Rhizosheath size of WT and *Stumpy* grown in sand and sand supplemented with lime (16 g CaCO_3_/ kg sand). **B** Root length of WT and *Stumpy* grown in sand and sand supplemented with lime. **C** Root length of WT and *Stumpy* grown by hydroponics in basal nutrient solution (0.5 mM CaCl_2_) or basal nutrient solution supplemented with 10 mM of various mineral nutrients. Data are expressed as a per cent of the seminal root length of WT. For all panels error bars indicate SE (*n* = 6 for **A** and **B**; *n* = 4 for **C** except for Ca(NO_3_)_2_ treatment where *n* = 5). The *Stumpy* line was backcrossed four times to WT, and *P* values were calculated by a two-way ANOVA for **A** and **B** or by Student’s *t* test for **C** comparing WT and mutant at each treatment. For **A** and **B** significant differences at *P* < 0.05 are indicated by different letters while for **C** significant differences at *P* < 0.001 are indicated (***)

A seedling with a large rhizosheath (soil adhering to roots) and short roots was identified while screening a mutagenised population of the wheat cv Westonia (Fig. 1). At early stages of growth (3 d), the mutation appeared to specifically affect roots as shoots did not show any obvious phenotype compared with WT plants (Fig. 1A). In view of its phenotype, the mutant was named *Stumpy*. The large rhizosheath could be attributed to longer root hairs than WT (Fig. 1B and D). When grown in a range of soils, the *Stumpy* phenotype varied from showing the typical *Stumpy* appearance to being indistinguishable from the WT (data not shown). However, the *Stumpy* phenotype was consistently present when grown in limed soil. We suspected that the liming was responsible for the mutant phenotype either through its pH effect or due to the high Ca^2+^ concentration. When grown in washed sand with no added nutrients, *Stumpy* did not show a phenotype that differed from WT (Fig. 2A, B). However, when CaCO_3_ was added to the sand, *Stumpy* showed the typical phenotype of a large rhizosheath and short roots (Fig. 2A, B).

Hydroponic culture is more precise than soil culture as a means of controlling mineral nutrient supply and pH. Seedlings were grown in hydroponic culture set at pH 6.0 and the phenotype was only apparent on seedlings grown in nutrient solutions that contained 10 mM Ca^2+^ as chloride or nitrate salts (Fig. 2C). The phenotype was not apparent on *Stumpy* seedlings grown in the same nutrient solutions that contained 10 mM KCl, 10 mM MgCl_2_ (Fig. 2C) or in NaCl concentrations ranging up to 100 mM (Fig. S1) indicating that the appearance of the phenotype was specifically associated with a high Ca^2+^ concentration and was not caused by a basic pH or a high ionic strength conferred by salts. To assess the effect of pH on the root phenotypes, we grew *Stumpy* and WT in nutrient solution over a wide range of pH values at both permissive (0.5 mM Ca^2+^) and non-permissive (10 mM Ca^2+^) conditions. Although pH affected both root and root hair elongation, the *Stumpy* phenotype was apparent only in the 10 mM Ca^2+^ treatments at all pH values (Fig. S2; pH range 5 to 9).

Analysis of F_2_ progeny from crosses of *Stumpy* with the WT indicated that the mutation initially appeared to be controlled by a single dominant gene with probabilities for segregation ratios that were not significantly different from a 3:1 ratio (161 mutant: 51 WT, *P* > 0.70 for a 3:1 ratio). Subsequent, more careful phenotyping indicated that two classes of F_2_ seedlings with the *Stumpy* phenotype could be identified. One class had a severe phenotype, whereas the other was intermediate between this class and wild type, suggesting that *Stumpy* is semi-dominant (Fig. S3). Because the phenotypes of heterozygous and homozygous *Stumpy* seedlings sometimes overlapped, seedlings classed as *Stumpy* comprised of both homozygotes and heterozygotes unless genotypes were confirmed in a subsequent generation.

### External Ca^2+^ concentration controls the severity of the Stumpy phenotype

**Fig. 3:**
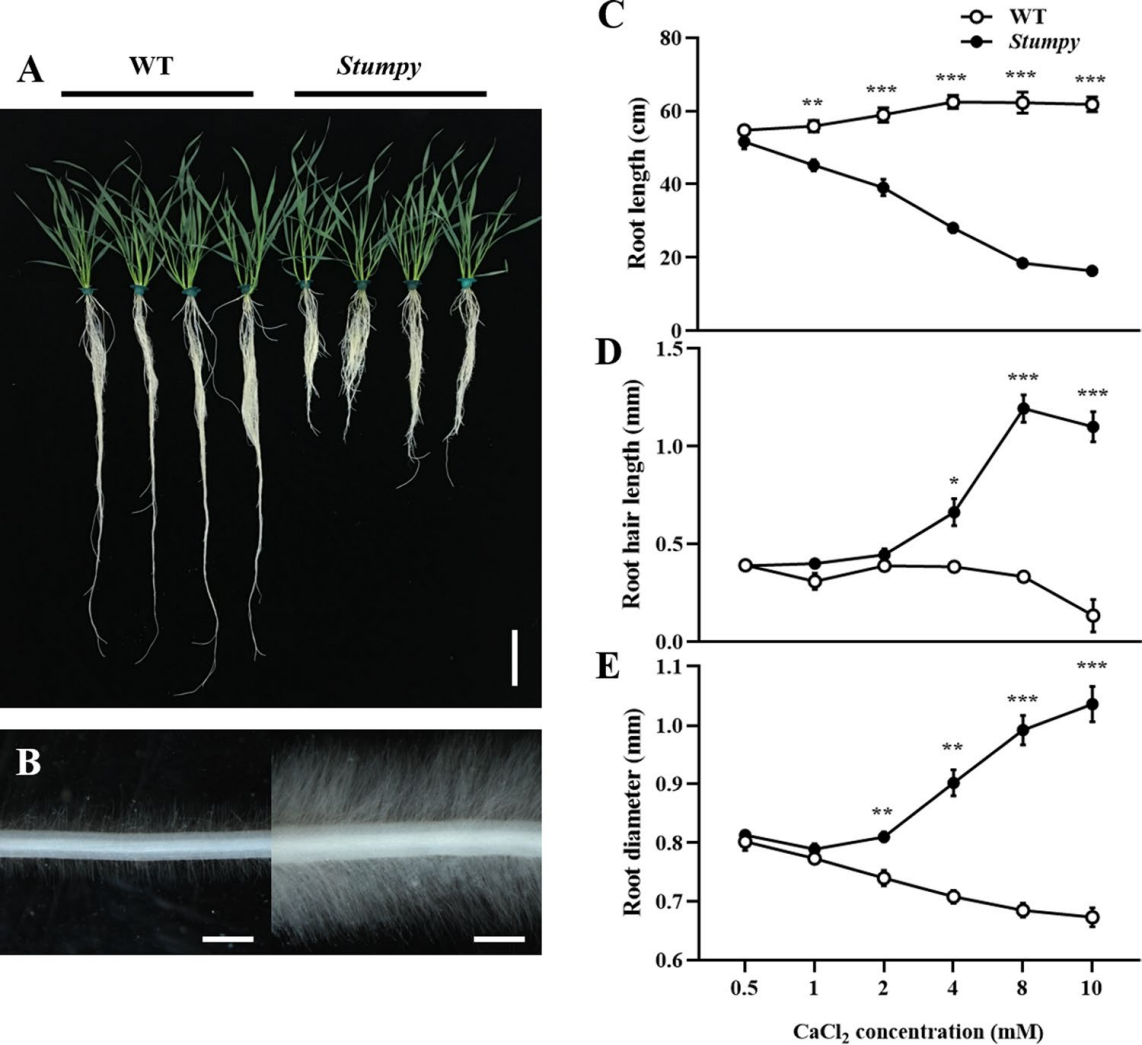
The severity of the *Stumpy* phenotype depends on the external Ca^2+^ concentration. **A** Whole plant (bar indicates 10 cm) and **B** root hair (bar indicates 1 mm) phenotypes of WT and *Stumpy* plants grown by hydroponics in nutrient solution supplemented with 4 mM CaCl_2_. **C** Length of longest root, **D** root hair length and **E** average root diameter of WT and *Stumpy* grown for 20 days by hydroponics supplemented with CaCl_2_ concentrations ranging from 0.5 to 10 mM. The *Stumpy* line was backcrossed four times to WT and for all panels error bars on symbols indicate SE (*n* = 6) and *P* values for significant differences were calculated by Student’s *t* test (* *P* < 0.05; ** *P* < 0.01; *** *P* < 0.001)

Hydroponic culture allowed us to compare *Stumpy* with WT plants across a range of Ca^2+^ concentrations. The root phenotypes were apparent on young seedlings grown in media containing high Ca^2+^ concentrations (3 days in soil or hydroponic culture) and remained on older seedlings after 20 days in hydroponic culture (Fig. 3). *Stumpy* seedlings were indistinguishable from wild type at 0.5 mM Ca^2+^ with shortening of roots appearing at Ca^2+^ concentrations of 1 mM and greater. Longer root hairs for *Stumpy* appeared above 2 mM Ca^2+^, while increased root diameter occurred above 1 mM Ca^2+^ (Fig. 3C-E, Fig.S4). As the Ca^2+^ concentration increased, so did the severity of the various *Stumpy* phenotypes.

Analysis of the shoot tissues indicated that the concentrations of most minerals were affected similarly in *Stumpy* and WT plants by the Ca^2+^ treatments. Only a few elements showed significant differences and these typically occurred at the high Ca^2+^ concentrations where the phenotypes were most severe (Fig. S5). For example, *Stumpy* accumulated less Mn at 10 mM Ca^2+^ and less Fe and *P* at Ca^2+^ concentrations above 2 mM. Although shoot Ca concentration responded strongly to the Ca^2+^ treatments as expected, mutant and WT did not differ at any of the treatments (Fig. S5A).

**Fig. 4:**
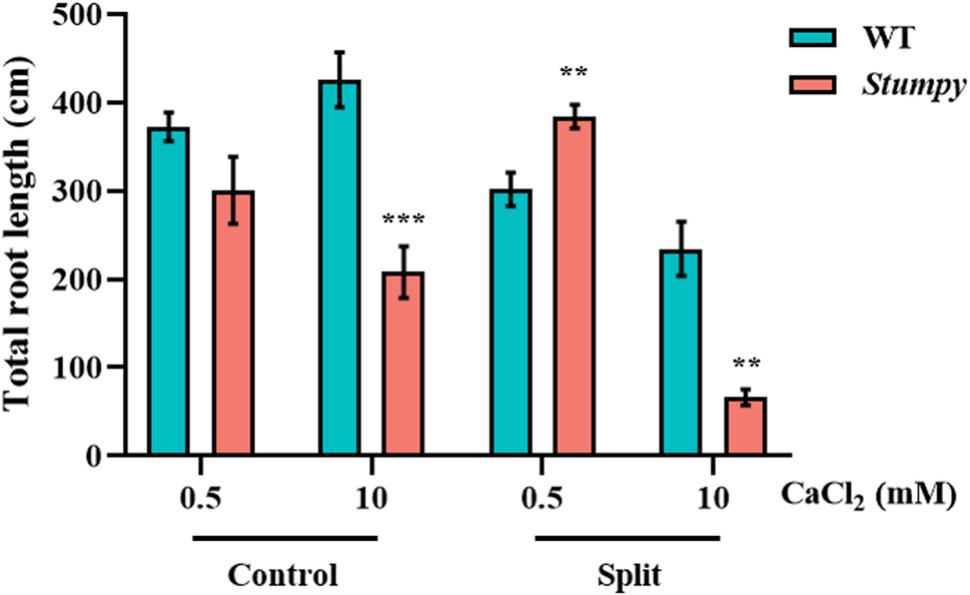
External Ca^2+^ controls the *Stumpy* phenotype. One part of a split root from each of WT and *Stumpy* were grown in low CaCl_2_ (0.5 mM) while the other part was grown in high CaCl_2_ (10 mM). None-split (Control) WT and *Stumpy* roots were grown entirely in either low or high CaCl_2_. MES was added to the solutions to stabilise the pH at 6.0. The *Stumpy* line was backcrossed four times to WT and total root length was measured after 14 days of growth. Error bars are the mean ± SE (*n* = 4) and asterisks indicate statistical significance comparing WT to *Stumpy* at each treatment as determined with Student’s *t* test (** *P* < 0.01; *** *P* < 0.001)

A split root experiment supported the notion that the external Ca^2+^ concentration controlled the *Stumpy* phenotype. Seedlings were grown in nutrient solution with one part of the root exposed to nutrient solution that contained 0.5 mM Ca^2+^, while the other part of the root was exposed to the same nutrient solution that contained 10 mM Ca^2+^. After 14 days of growth, only the side of the root exposed to 10 mM Ca^2+^ was significantly shorter for *Stumpy* compared to WT grown in the same containers, whereas *Stumpy* appeared to compensate for the shorter roots in high Ca^2+^ by having longer roots than WT on the side exposed to 0.5 mM Ca^2+^ (Fig. 4). These results indicate that the phenotype is not induced by a systemic signal in the seedlings but instead requires the roots to be in direct contact with a high Ca^2+^ concentration.

The *Stumpy* mutation specifically affected the roots in short-term exposures to high Ca^2+^ because no phenotypes were detected in the shoots. Shoot growth, however, was reduced after longer exposures to the high Ca^2+^ concentrations (Fig. S6) but this was likely a consequence of having shortened roots for an extended time. When grown to maturity in potting mix with a permissive Ca^2+^ concentration, plant height, tiller number and grain yield of *Stumpy* were similar to WT (Fig. S7).

### High Ca^2+^ concentration shortens root cells in the Stumpy mutant

**Fig. 5:**
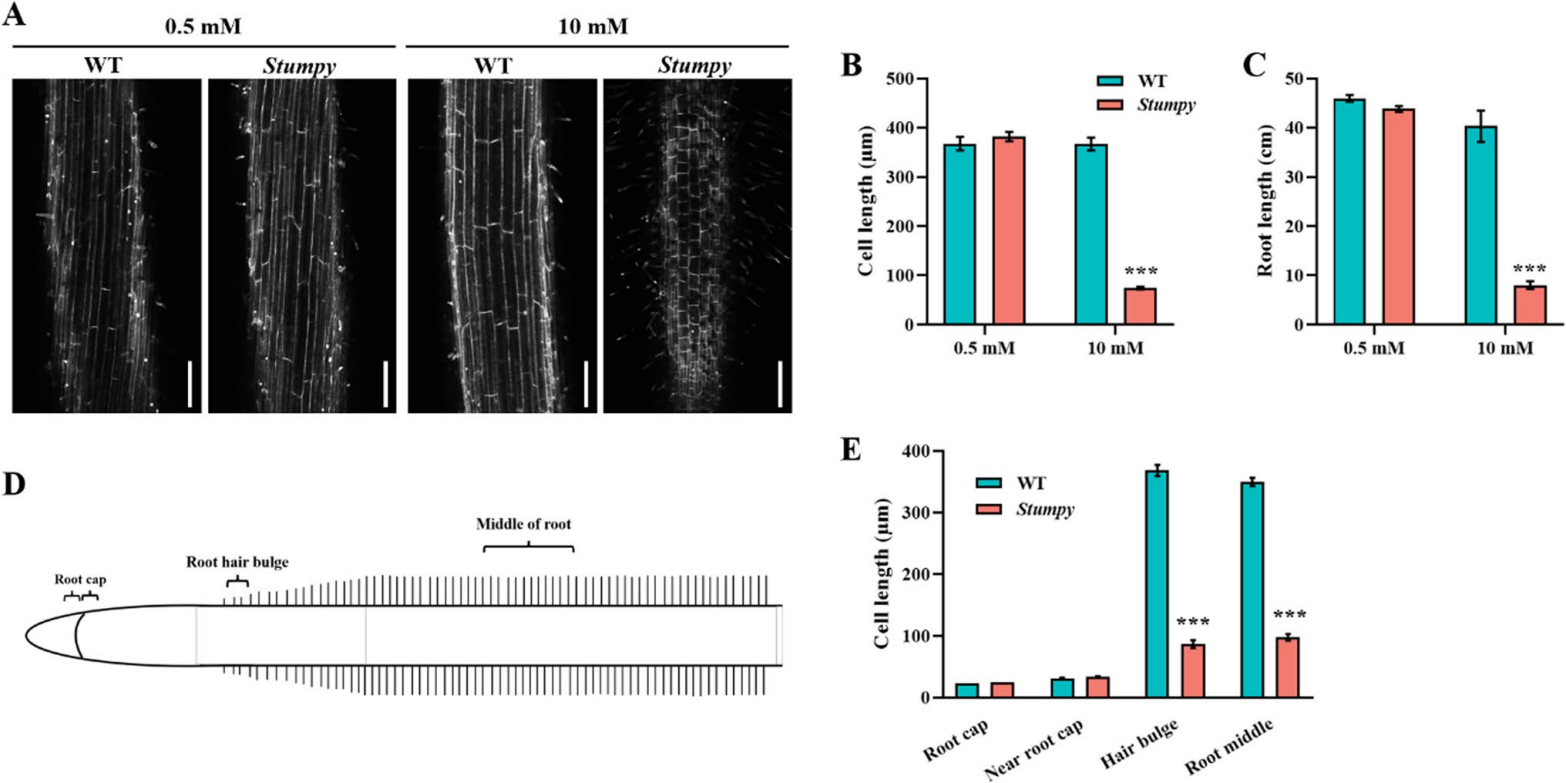
*Stumpy* has shortened root cells at non-permissive (high) Ca^2+^ concentrations. **A** Phenotype of root cells of WT and *Stumpy* grown in 0.5 and 10 mM CaCl_2_ in a region of the root approximately where hairs had started to develop (bars indicate 200 μm). **B** Cell length (excluding hairs of epidermal cells) and **C** root length of WT and *Stumpy* grown in 0.5 and 10 mM CaCl_2_. **D** Schematic diagram of a root showing the regions where cell length was measured with meristematic cells located in the region near the root cap. **E** Cell length in 4 regions of the root comparing *Stumpy* and WT grown for 16 days by hydroponics. The *Stumpy* line was backcrossed four times to WT, and MES at 1 mM was added to all nutrient solutions to stabilise the pH at 6.0. For **B**, **C** and **E** asterisks above the bars indicate WT and *Stumpy* were significantly different from one another as determined by Student’s *t* test (***; *P* < 0.001). Error bars denote the SE (*n* = 4)

The short roots of *Stumpy* grown under non-permissive Ca^2+^ concentrations could be attributed to shortened cells. At a permissive Ca^2+^ concentration, cell length of *Stumpy* and WT did not differ from one another in a region of the root where cells had reached the end of elongation (Fig. 5A-C). The differences in root length at high Ca^2+^ could be attributed entirely to the differences in cell lengths since the root cells in WT were approximately fivefold longer than those in *Stumpy* which reflects the relative differences in cell length (Fig. 5B, C). The length of cells in the meristematic region of the developing root was similar for *Stumpy* and WT but differed markedly at the proximal end of the elongation zone and remained different in mature cells (Fig. 5D, E). It appeared that the *Stumpy* mutation specifically restricted elongation of root cells at the non-permissive Ca^2+^ concentrations. However, by contrast, at the same non-permissive Ca^2+^ concentrations, root hairs were elongated in *Stumpy* indicating a gene that has opposing effects on the different cellular components.

### Monosaccharide linkage composition

**Fig. 6:**
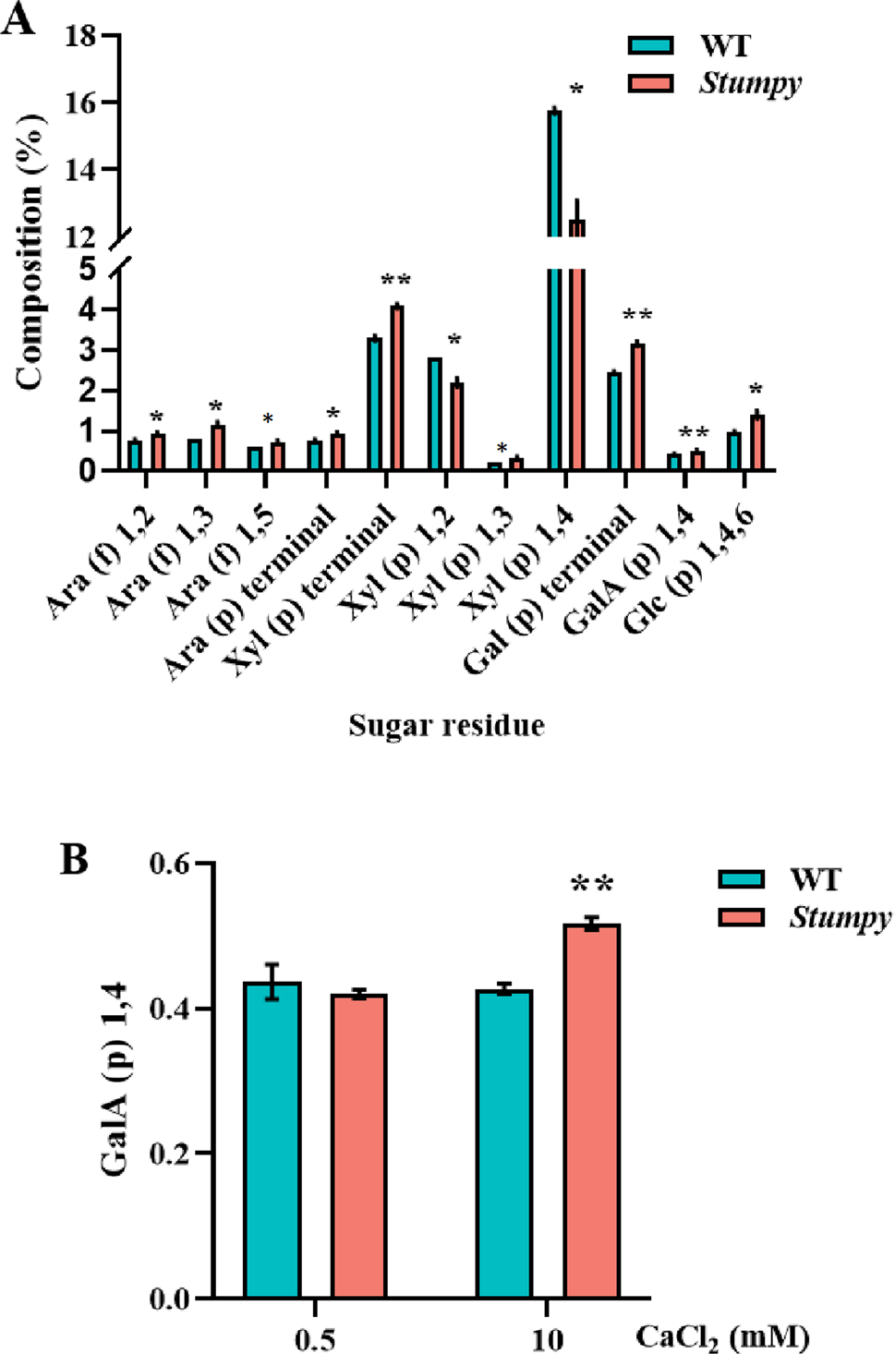
Monosaccharide linkage analysis of WT and *Stumpy* roots grown in 10 mM Ca^2+^. **A** The composition of monosaccharides (excluding GalA(p) 1,4-) that differed in root cell walls of WT and *Stumpy* grown in 10 mM Ca^2+^. The complete monosaccharide linkage analysis is shown in Table S2. **B** The HG composition, as shown by GalA(p) 1,4-, of WT (green bars) and *Stumpy* (magenta bars) roots grown in 0.5 mM Ca^2+^ and 10 mM Ca^2+^. The *Stumpy* line was backcrossed four times to WT and MES at 1 mM was added to stabilise the pH of solutions at 6.0. Error bars indicate SE and asterisks above bars indicate significant differences between genotypes as determined by Student’s *t* test (*n* = 3; * *P* < 0.05; ** *P* < 0.01)

The mechanism underlying the *Stumpy* phenotypes needs to explain two observations: (i) the conditional nature of the mutant where the phenotypes of short roots and long root hairs become apparent only at a high external Ca^2+^ concentration, and (ii) the *Stumpy* phenotype only occurring on those roots that are directly in contact with a high Ca^2+^ concentration. To evaluate the idea that cell wall composition could be controlling the *Stumpy* phenotype, we isolated cell walls of roots grown under permissive (0.5 mM) and non-permissive (10 mM) Ca^2+^ concentrations to analyse the monosaccharide linkage composition as a means of deducing cell wall polysaccharide structures. Minor differences in monosaccharide linkage compositions of root cell walls between WT and *Stumpy* were apparent for plants grown at 0.5 mM Ca^2+^ (Table S2), whereas many more differences were apparent for plants grown in 10 mM Ca^2+^ (Fig. 6A; Table S2). Of particular interest was (1,4)-linked GalA(p) content as a monosaccharide linkage indicative of the homogalacturonan (HG) content which could help explain the Ca^2+^ dependence of the *Stumpy* phenotype. Homogalacturonan is able to bind Ca^2+^ causing cell walls to stiffen (Proseus and Boyer 2006) and was elevated in *Stumpy* compared to WT but only for plants grown at high external Ca^2+^ (Fig. 6B). However, HG content was low, as expected for wheat, under both Ca^2+^ treatments, whereas xylans were much more abundant with larger differences between genotypes. Whereas no differences from linkage analysis were found at 0.5 mM Ca^2+^, some of the xylans were elevated, while others were decreased when *Stumpy* was compared to WT at 10 mM Ca^2+^ (Fig. 6A; Table S2).

### Auxin

The phenotype of WT wheat roots exposed to a high external concentration of auxin superficially phenocopied that of *Stumpy* exposed to high Ca^2+^ concentration by having short roots associated with long hairs (Fig. S8A). We explored the possibility that auxin was central in conferring the *Stumpy* phenotype by assessing the responses of the genotypes to a range of external auxin concentrations and by measuring internal auxin concentration in roots. The genotypes showed a similar sensitivity towards application of an external auxin analog in inhibiting root growth for plants grown at 0.5 mM Ca^2+^ (Fig. S8B). Furthermore, concentrations of auxins in roots and shoots were similar for both genotypes at both permissive and non-permissive Ca^2+^ concentrations (Fig S8C and D). Indole acetic acid (IAA) was the most abundant auxin of both roots and shoots, whereas auxin conjugated to various amino acids was at lower concentrations and subject to greater variability in the assay. These observations indicated that *Stumpy’s* response to external auxin and its internal auxin concentration did not appear to be grossly perturbed when compared to WT.

### The Stumpy mutation maps to chromosome 7B

**Fig. 7:**
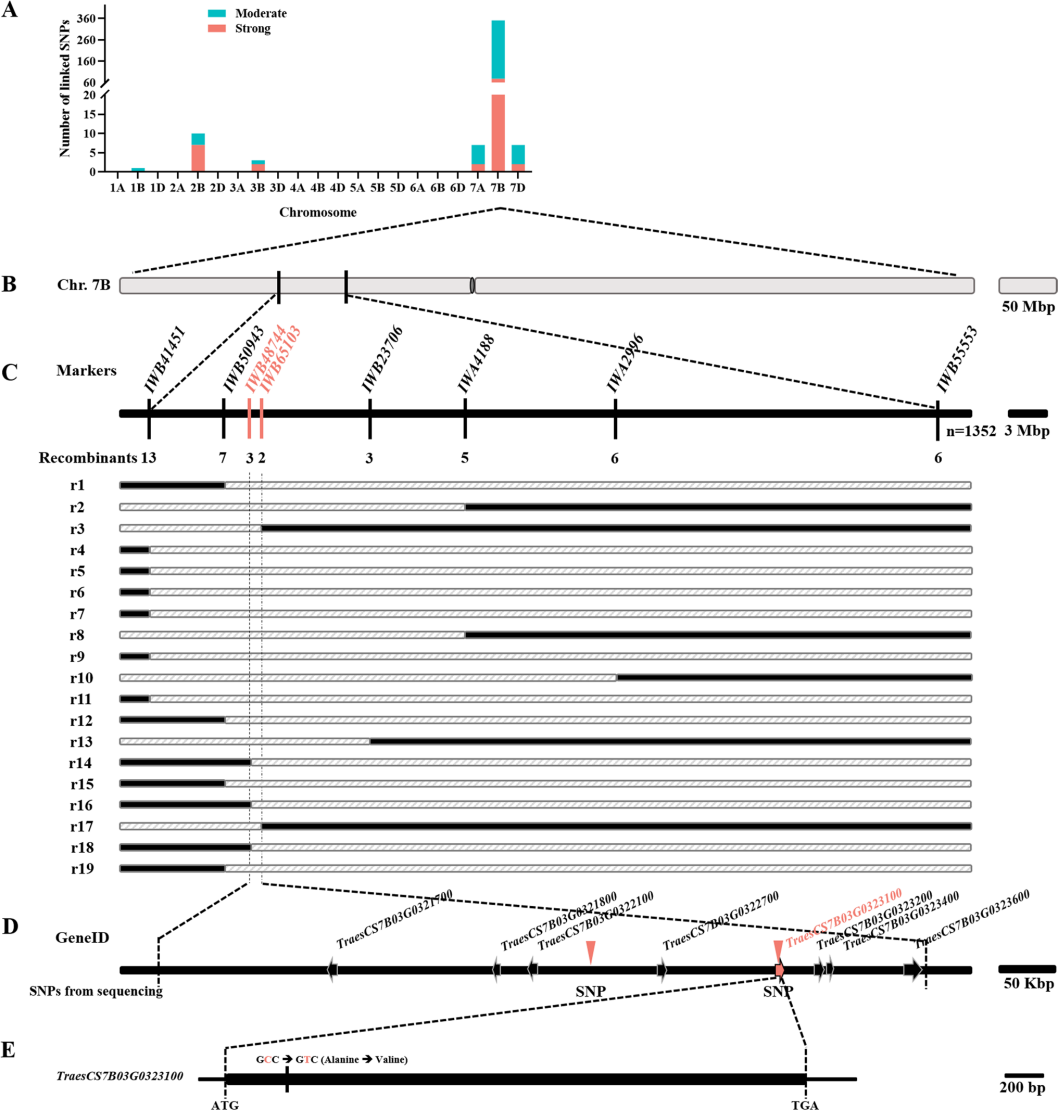
The *Stumpy* mutation is located on the short arm of chromosome 7B and sequencing identifies gene *TraesCS7B03G0323100* as a candidate*.* **A** The number and chromosomal locations of SNPs linked to the *Stumpy* mutation on the various chromosomes of hexaploid wheat indicating those with moderate and strong linkage. **B** Schematic of the whole 7B chromosome indicating the locations of SNP markers found to flank the *Stumpy* mutation (see Table S1 for primer sequences used for KASP assay). **C** Schematic of the region defined by flanking markers at **B** with additional SNP markers (vertical lines) used to fine map the *Stumpy* mutation. The numbers indicate the number of recombinant seedlings identified with each marker. Markers in magenta indicate the closest flanking markers as determined by analysis of DNA from recombinant seedlings (r1 to r19) shown below the diagram of the chromosomal region. The solid and hatched lines indicate where chromosomes have recombined as determined by marker analysis. **D** The region where the *Stumpy* mutation was located by fine-mapping is shown with the two closest flanking markers that encompass about 0.69 Mbp based on the Chinese Spring physical map v2.1. Sequencing of purified 7B chromosomes identified two C to T transitions in *Stumpy* within this region denoted by the magenta arrows. One of the SNPs is located within the coding region of *TraesCS7B03G0323100* (magenta gene) while the other SNP is located in an intergenic region of chromosome 7B. **E** Schematic of *TraesCS7B03G0323100* showing the location of the mutation resulting in an alanine to be substituted by valine in the predicted TraesCS7B03G0323100 protein

To map the mutation, we generated F_3_ lines homozygous for WT and *Stumpy* derived from F_2_ seedlings of the cross between *Stumpy* in the cv Westonia genetic background to WT in the cv Chara genetic background. After verifying phenotypes in the F_3_ generation, we used a 90 K SNP chip to identify markers that co-segregated with the *Stumpy* phenotype. From this analysis we established that the *Stumpy* mutant was located on chromosome 7B (Fig. 7A). The 7B location was confirmed by KASP markers on segregating F_2_ and F_3_ populations derived from the *Stumpy* by cv Chara cross. SNP polymorphisms between cv Westonia and Chara were used to map the *Stumpy* mutation to a region of about 0.69 Mbp on the short arm of chromosome 7B (Fig. 7A-C). Finer mapping was hampered by a lack of suitable polymorphic markers, so we embarked on a sequencing strategy to identify possible causative mutations.

### Sequencing of chromosome 7B identifies a candidate gene for the Stumpy mutant

Whole genome sequencing is generally out of reach for most laboratories to routinely clone genes based solely on a mutant phenotype from hexaploid wheat. However, methodologies based on purification of specific chromosomes and subsequent whole chromosome sequencing have been successfully used to identify causative mutations (Sánchez-Martín et al. 2016). For *Stumpy,* we purified chromosome 7B by flow sorting of backcrossed germplasm comparing the 7B sequence of a *Stumpy* line to the WT that was used as the recurrent parent. Comparison of sequences in the ~ 0.69 Mbp region defined by the two closest flanking SNP markers identified two C to T transitions consistent with the action of Na azide as a mutagen (Fig. 7D). One of the transitions was located in an intergenic region of chromosome 7B approximately 45 Kb upstream of the transcriptional start site of gene *TraesCS7B01G124400* encoding an uncharacterised protein. The other transition was located in the coding region of gene *TraesCS7B03G0323100* and caused a missense mutation changing the alanine at position 113 of the protein to a valine (Fig. 7D, E). The protein encoded by *TraesCS7B03G0323100* is of unknown function that shows some similarity to an embryogenesis transmembrane protein and has 5 PGG domains (pfam 13,962) of unknown function and one epiglycanin-TR domain (pfam 05647) which is a tandem repeating region of the membrane-bound protein mucin (Itoh et al. 2008). *TraesCS7B03G0323100* is predicted to encode a large membrane protein of about 100 KDa with 17 to 20 transmembrane domains and is most likely located on the plasma membrane with the altered amino acid occurring in the second predicted transmembrane domain (Fig. S9). *TraesCS7B03G0323100* was expressed in WT and *Stumpy* roots to the same level at both permissive (0.5 mM) and non-permissive (10 mM) Ca^2+^ concentrations consistent with the mutation in the coding region of the gene not affecting level of expression (Fig. S10).

### Screen for independent mutants

To establish whether the mutation in *TraesCS7B03G0323100* was causative, we undertook a screen of a Cadenza TILLING population and searched for independent mutants with the same or similar phenotypes as *Stumpy*. First, we searched the online database and targeted lines with mutations in *TraesCS7B03G0323100*. Searching the database, we focussed on lines that caused a change in amino acid or possessed a stop codon but none caused the specific change of alanine to valine as found in *Stumpy*. There was good agreement between the quantified rhizosheath screen undertaken in Dundee, Scotland and the visual screen undertaken in Canberra, Australia (Fig. S11A) with only 4 out of 29 lines having markedly different rankings. Many of the lines with large rhizosheaths had similar root lengths to WT Cadenza0000, indicating that a large rhizosheath is not always associated with a short root. Although some of the TILLING lines selected for assay had larger rhizosheath sizes than WT (Fig. S11A), none were conditional on the Ca^2+^ concentration as found for *Stumpy*. Nevertheless, we reasoned that different mutations in the same gene might confer different phenotypes associated with roots. Line Cadenza0798 with a stop codon within the coding region of *TraesCS7B03G0323100* was not screened at Dundee but was subsequently analysed in Canberra and found to possess a smaller rhizosheath than WT Cadenza0000 (Fig. S12A). Root hair phenotypes of the F_1_ and parental genotypes of a cross between Cadenza0798 and Cadenza0000 suggested the mutation was recessive since the F_1_ had long root hairs (Fig. S12B-D). Sequence analysis of eight lines identified five having mutations in the expected location of *TraesCS7B03G0323100* (Table S3), whereas the altered rhizosheath sizes of other sequenced lines must have been caused by a different gene.

## Discussion

The *Stumpy* phenotype in wheat is caused by a mutation in a gene that plays a key role in defining root morphology. The mutant is conditional on the external Ca^2+^ concentration and under non-permissive conditions has greatly shortened roots with elongated root hairs. The short roots of *Stumpy* can be entirely explained by the effect of Ca^2+^ concentration on elongation of root cells (Fig. 5). The effects of the mutation on root hair elongation and root cell elongation point to a mechanism with opposing effects on hairs and the basal body of root cells. Although apparently contradictory phenotypes, the effects on root hair elongation and cell expansion might have a common basis. The inhibition of cell elongation suggests that cell walls constrain cells from expanding resulting in increased turgor pressure which in turn could drive the elongation of hairs. Observations to support this notion comes from a study of wheat germplasm varying for vigour where cell size and root hair length were inversely correlated (Hendriks et al. 2022). The thicker roots of Stumpy grown with high Ca^2+^ (Fig. 3E) is also consistent with the notion of increased turgor pressure within root cells where cells in *Stumpy* restricted in elongating longitudinally, bulge in a radial direction. Furthermore, when the total cellular volume of trichoblasts is estimated (cell body plus hair) for roots grown in high Ca^2+^, it is approximately equal to the total cellular volume of trichoblasts at low Ca^2+^ (Supplemental Text S1) suggesting that cell volume is conserved for trichoblasts with the hairs acting as a “release valve” for increased turgor.

The *Stumpy* phenotype is unique in that there are no reports to date for any plant species of dominant or semi-dominant root mutants that are conditional on Ca^2+^ concentrations. The sterol methyl transferase 1 (*smt1*) mutants of Arabidopsis are the most similar to *Stumpy* in that a phenotype of shortened roots occurs specifically when seedlings are grown in the presence of high Ca^2+^ concentrations (Diener et al. 2000). However, the mutants are recessive and the short roots are not accompanied by long root hairs. Furthermore, epidermal cells of *smt1* appear swollen and distorted at high Ca^2+^ concentrations, symptoms indicative of Ca stress that are not apparent on *Stumpy* at non-permissive Ca^2+^ concentrations. Similarly, an Arabidopsis mutant of a cyclic nucleotide gated channel is recessive and specifically hypersensitive to high Ca^2+^ concentrations such that at high Ca^2+^ concentrations overall plant growth is reduced and not just the roots (Chan et al. 2003). Although growth of *Stumpy* shoots was inhibited by high Ca^2+^, this was only apparent after prolonged growth (Fig. S6).

Our initial idea to explain how the mutant is conditional on high external Ca^2+^ concentrations was based on observations that cell wall expansion in the algae *Chara corallina* is controlled by external Ca^2+^ concentrations (Proseus and Boyer 2006). In that system, expansion of cells appeared to be controlled by the ability of Ca^2+^ to cross-link to pectin molecules through the homogalacturonan (HG) residues. The mechanism requires Ca^2+^ to interact with de-methylesterified HG as it is the free carboxyl groups that bind to Ca^2+^. This is an attractive idea to explain the reliance of the *Stumpy* phenotype on a high external Ca^2+^ concentration. It was possible that pectin concentration in *Stumpy*, particularly the demethylated form, was elevated such that at high Ca^2+^ it was able to form “egg-box” structures (Peaucelle et al. 2012) to restrict cell elongation. A recent report found that the egg-box structures with Ca^2+^ exist in plants and can control cell elongation (Temple et al. 2022). To determine if cell wall composition was perturbed in *Stumpy*, an analysis of monosaccharide linkages identified numerous differences apparent between WT and *Stumpy* when grown in 10 mM Ca^2+^ (Fig. 6A). Homogalacturonan in its de-methylesterified form is the component of pectin able to bind Ca^2+^ and although HG content was elevated in cell walls of *Stumpy* roots compared to WT (Fig. 6B), the increase was small and the total content low compared to HG abundance in cell walls of dicotyledons. By contrast, a study in Arabidopsis has shown it is the de-methylesterified form of HG that drives cellular expansion independent of turgor pressure which would appear to counter the Ca^2+^ egg-box idea (Haas et al. 2020). Consistent with this concept, a study of Arabidopsis hypocotyls showed that formation of methylesterified HG was induced by auxin and explained how the hypocotyl formed its hook structure by restricting elongation on the side of the tissue that accumulated methylesterified HG (Jonsson et al. 2021). The degree of esterification of HG between WT and *Stumpy* was similar at both permissive and non-permissive conditions so does not implicate either mechanism over the other (egg-box versus methylesterified) as the reason for restricted cell elongation in *Stumpy* roots. However, it should be noted that much of what is known about cellular expansion in plants is derived from studies on dicotyledons where pectin is a major component of cell walls. Although the low HG content of monocotyledons appears to be problematic in evoking a role for HG in cell expansion, Haas and Peaucelle (2021) argue that recycling of HG might be elevated in monocotyledons compared to dicotyledons and could yet play a central role in cell expansion. It should also be noted that our carbohydrate analysis used whole roots that would have masked larger differences that could have occurred at the cellular level in specific regions of the root. Studies with antibodies showed that elongating cells of maize had de-methylesterified HG, whereas methylesterified HG was primarily located in walls of the small cells of the meristem and largely absent from elongating cells (Petrova et al. 2021). Furthermore, although our focus was on HG in pectin as a key molecule involved in cell expansion, other cell wall components were altered in the mutant compared to WT grown with a high Ca^2+^ concentration (Fig. 6A) and the overall remodelling of cell wall components could conceivably be required to generate the *Stumpy* phenotype. In particular, xylan residues were much more abundant than HG and the various linkages showed greater differences between genotypes at 10 mM Ca^2+^ (Fig. 6A; Table S2). The branching of xylans is implicated in cell wall strength and extensibility through polymer interactions although how they could interact with Ca^2+^ treatments is unclear (Tryfona et al. 2023). A study using molecular dynamics simulations found that Ca^2+^ could cross-link between glucuronic acid substitutions of neighbouring xylan chains to stabilise binding although these structures have not yet been verified *in planta* (Pereira et al. 2017).

Internal Ca^2+^ concentrations are part of the signal transduction pathway for auxin responses that modify roots but typically Ca^2+^ concentrations in the cytosol act in the sub-micromolar range (Dodd et al. 2010). The *Stumpy* phenotype under non-permissive conditions is not a phenotype consistent with Ca^2+^ toxicity on roots and it appears that the mutation has changed the function of a protein due to the mutation. The alanine to valine substitution present in the second transmembrane domain of the protein predicted to be encoded by *TraesCS7B03G0323100* is a good candidate to be the causative mutation. There are numerous examples of alanine to valine substitutions resulting in dominant or semi-dominant mutations that alter protein function including examples of substitutions in transmembrane domains of membrane-bound proteins. For example, alanine to valine substitutions in a pore domain of a Na^+^ channel of cockroaches confer Na^+^ channel resistance to DDT (Chen et al. 2017). In humans the often fatal long-QT syndrome results in cardiac arrhythmias in otherwise healthy young people and is typified by autosomal dominant mutations. One of the earliest causative mutations identified in the K^+^ channel underlying long-QT is due to an alanine to valine substitution in a transmembrane spanning domain that in humans often results in early death (Li et al. 1998; Wang et al. 1996). In Arabidopsis, an alanine to valine substitution in a protein kinase causes stomata to remain open more often than wild type (Horak et al. 2016). The mutation is dominant but in this case the protein is not membrane-bound. How the *Stumpy* mutation acts to alter cell expansion remains unknown. Initially we thought the gene could encode a protein that is a receptor or transporter of Ca^2+^ with the mutation altering its function. The protein encoded by *TraesCS7B03G0323100* does not have conserved domains typical of Ca^2+^ transport or Ca^2+^-binding proteins and did not show significant homology to proteins of known function although it had stronger similarities to similar proteins in monocotyledons than in dicotyledons (data not shown).

Our search of the Cadenza TILLING population did not identify lines with the same conditional phenotype as *Stumpy* although it identified lines with larger rhizosheaths than the control WT. Unlike a loss-of-function mutation, a gain-of-function mutation like *Stumpy* may need specific changes to the protein and none of the Cadenza lines possessed the exact same mutation as *Stumpy*. For example, the dominant *AUXIN/INDOLE-3-ACETIC ACID* gain-of-function mutants of many plants are mutated in a highly conserved region of the gene encoding five amino acids, whereas mutations in other regions of the gene do not confer a similar dominant phenotype (Overvoorde et al. 2005). Indeed, Cadenza lines that were confirmed to carry a mutation in *TraesCS7B03G0323100* possessed the mutation distant from the one found in *Stumpy* (Table S3). Interestingly, line Cadenza0798 possessed a stop codon in the coding region of *TraesCS7B03G0323100*, had a small rhizosheath and was a recessive mutation (Fig. S12). Although the rhizosheath phenotypes of the Cadenza mutants were not conditional on the Ca^2+^ concentration, the screens showed that variation of rhizosheath size, and by implication root hair length, exists within the TILLING population. Importantly, for several of the mutants the larger rhizosheaths were not associated with shortened roots as found for *Stumpy*. This indicates that the TILLING population is a useful resource for identifying lines with long root hairs while maintaining root length, attributes of value for improving nutrient and water uptake efficiency in soil-grown plants. For some of the Cadenza mutants where we were able to generate KASP markers, F_2_ populations generated by crossing the mutant to WT Cadenza were screened to assess if the rhizosheath phenotype co-segregated with the marker. However, this proved inconclusive as the phenotypes of the Cadenza TILLING mutants were subtle compared to *Stumpy*. For example, rhizosheath sizes of the Cadenza mutants were at best only about 1.5-fold of WT (Fig. S11) and there was a degree of variability associated with the rhizosheath assay for individual seedlings. Furthermore, the TILLING population was generated by a heavy mutagenesis so any given TILLING line has thousands of mutations with each line having a unique set of mutations. When a segregating F_2_ population is generated then many of the mutations would be segregating. We can expect that a proportion of the mutations would affect root phenotypes likely resulting in genetic variability between individual seedlings affecting the rhizosheath phenotype. Figure S13 illustrates this point with one of the TILLING lines used as a parent in a cross to WT Cadenza0000 and although mean values for rhizosheath sizes of an F_2_ population appear consistent with co-segregation, values for individual seedlings did not show a clear co-segregation. Therefore, the mutation in *TraesCS7B03G0323100* might still have been responsible for the phenotype but the variability due to the assay itself along with genetic variability of segregating lines means the data are inconclusive regarding the co-segregation analysis.

*Stumpy* is a valuable mutant for studying the physiology and biochemistry of cell expansion in a monocotyledonous species of agricultural significance. While *Stumpy* itself has limited direct value in agriculture, understanding the mechanisms behind cell expansion and root hair elongation could form the basis for modifying these root traits. Furthermore, alleles of *Stumpy* may exist that dissociate the long hairs from short roots as well as the conditional nature of the phenotype as suggested by identification of the various Cadenza TILLING mutants. Further work will be aimed at determining whether the mutation in *TraesCS7B03G0323100* is the cause of the *Stumpy* phenotype. This could be undertaken by expressing the mutated and WT versions of the gene in wheat but could be confounded by the presence of the endogenous WT version of the gene. A more concise strategy would be to specifically replicate the C to T transition in *TraesCS7B03G0323100* of hexaploid wheat using clustered regularly interspaced short palindromic repeats (CRISPR)/CRISPR associated protein (CAS) technology. CRISPR/CAS is more generally applied for generating knock outs of specific genes but is increasingly being applied to generate gain-of-function mutants in plants and is becoming a precise tool for generating mutants in hexaploid wheat (Gaillochet et al. 2023; Ni et al. 2023).

### Supplementary Information

Below is the link to the electronic supplementary material.

Supplementary file1 (DOCX 7667 KB)

## Data Availability

I confirm that materials described in the manuscript (e.g. mutants, genetic stocks, novel resistance sources, transgenic plants, vectors, antibodies, enzymes, primer sequences or software) will be freely available to any researcher wishing to use them for non-commercial purposes.
