# Supplementary material for: A conditional mutation in a wheat (Triticum aestivum L.) gene regulating root morphology
Source: Theor Appl Genet. 2024 Feb 12;137(2):48. doi: 10.1007/s00122-024-04555-7 (PMC10861616; doi:10.1007/s00122-024-04555-7)
Supplement: Supplementary file 1 — Supplementary file1 (DOCX 7667 KB) [file 122_2024_4555_MOESM1_ESM.docx]

| **Supplemental Data**  **Table S1.** Marker information and primer sequences used for KASP assays of SNPs and primer sequences used for qRT-PCR of *TraesCS7B03G0323100.* | | | |
| --- | --- | --- | --- |
| Marker ID | SNP name* | Forward primer (5'-3')** | Reverse primer (5'-3') |
| IWA7326 | wsnp_Ku_c8497_14429303 | GAAGGTGACCAAGTTCATGCTGGACAAGGCATGAGGAAGTTT | ACACCTGGAACATTTGACCC |
|  |  | GAAGGTCGGAGTCAACGGATTGGACAAGGCATGAGGAAGTTC |  |
| IWB41451 | Kukri_c1576_1469 | GAAGGTGACCAAGTTCATGCTCTAGAACAAGATGCAGTGTTTCA | CTGAGGCAGGTAATGTTGTATATG |
|  |  | GAAGGTCGGAGTCAACGGATTCTAGAACAAGATGCAGTGTTTCG |  |
| IWA6661 | wsnp_Ku_c18780_28136150 | GAAGGTGACCAAGTTCATGCTTGCTAGAGAAGAAGGGTGGT | CCAACAGCAAGCAACAACG |
|  |  | GAAGGTCGGAGTCAACGGATTTGCTAGAGAAGAAGGGTGGC |  |
| IWB24159 | Excalibur_c24453_670 | GAAGGTGACCAAGTTCATGCTGACAAAAGCAGAAGATCTAACATGT | ACTGAATTCGTAGCAATATAGCATC |
|  |  | GAAGGTCGGAGTCAACGGATTGACAAAAGCAGAAGATCTAACATGC |  |
| IWB74343 | tplb0028n22_365 | GAAGGTGACCAAGTTCATGCTCGGCCTCGACCAACCCTT | GGCAGCTTTGAATATGTCACTC |
|  |  | GAAGGTCGGAGTCAACGGATTCGGCCTCGACCAACCCTC |  |
| IWB46358 | Kukri_c53648_585 | GAAGGTGACCAAGTTCATGCTCACACCCGACTTAATGTACCT | CTTCTAGATTGATGACGAGCAATTT |
|  |  | GAAGGTCGGAGTCAACGGATTCACACCCGACTTAATGTACCG |  |
| IWA3174 | wsnp_Ex_c2857_5275817 | GAAGGTGACCAAGTTCATGCTCCGTCTGATGAATTCTTGGCA | AGCTTCAACAAAATAGACGATTCTT |
|  |  | GAAGGTCGGAGTCAACGGATTCCGTCTGATGAATTCTTGGCG |  |
| IWA2996 | wsnp_Ex_c25755_35018040 | GAAGGTGACCAAGTTCATGCTTTGAGCTATCTGATATCACAGGTT | GCTGAGATGCATGCCTAATG |
|  |  | GAAGGTCGGAGTCAACGGATTTTGAGCTATCTGATATCACAGGTC |  |
| IWB58816 | RAC875_c52266_76 | GAAGGTGACCAAGTTCATGCTTCAGCACCAGCCACTGCA | TCCTGGAGTCAATCTTTTACTGTT |
|  |  | GAAGGTCGGAGTCAACGGATTTCAGCACCAGCCACTGCG |  |
| IWA2964 | wsnp_Ex_c2539_4733110 | GAAGGTGACCAAGTTCATGCTAGCTGCTCGACTCGGCTT | TTGAGGAAGTACACCTATGGCAAG |
|  |  | GAAGGTCGGAGTCAACGGATTAGCTGCTCGACTCGGCTC |  |
| IWA311 | wsnp_BE498662B_Ta_2_1 | GAAGGTGACCAAGTTCATGCTGGCACCCATATCATAATATCAGCA | TCGCTAAATTTCTTGGGCGG |
|  |  | GAAGGTCGGAGTCAACGGATTGGCACCCATATCATAATATCAGCG |  |
| IWB22804 | Excalibur_c16568_1190 | GAAGGTGACCAAGTTCATGCTAACAACTGGTGAACTGAGACT | CATCTGACCAAACAAACTTCATACA |
|  |  | GAAGGTCGGAGTCAACGGATTAACAACTGGTGAACTGAGACC |  |
| IWB55553 | RAC875_c23724_118 | GAAGGTGACCAAGTTCATGCTTGCCAGGTTGCTACATTTCTA | GAACGACGAGACTGGCTCTA |
|  |  | GAAGGTCGGAGTCAACGGATTTGCCAGGTTGCTACATTTCTG |  |
| IWB7824 | BS00031141_51 | GAAGGTGACCAAGTTCATGCTGCATCCATTTTCACATCTGAAACAT | CGCGGTAACATATGTTTAAACAAC |
|  |  | GAAGGTCGGAGTCAACGGATTGCATCCATTTTCACATCTGAAACAG |  |
| IWA1543 | wsnp_Ex_c11860_19030807 | GAAGGTGACCAAGTTCATGCTCAAGCCATGCTCATGAAATTTCTA | TCCCAAATAATATTCACCCATGGT |
|  |  | GAAGGTCGGAGTCAACGGATTCAAGCCATGCTCATGAAATTTCTG |  |
| IWB1376 | BobWhite_c20290_226 | GAAGGTGACCAAGTTCATGCTCGGTGAAGCCACTACCTTGTT | TTTTGCGGTGAGGTGTGTCT |
|  |  | GAAGGTCGGAGTCAACGGATTCGGTGAAGCCACTACCTTGTC |  |
| IWB48744 | Kukri_rep_c102204_68 | GAAGGTGACCAAGTTCATGCTCCATCTTCCGTACAACATTCTACA | GCCACTTGCCATTTTACGAC |
|  |  | GAAGGTCGGAGTCAACGGATTCCATCTTCCGTACAACATTCTACG |  |
| IWB61734 | RAC875_rep_c107786_249 | GAAGGTGACCAAGTTCATGCTTGATTAACAGCTCCCTGAAGAAT | AAAGGGAGTGGCTGGAGATC |
|  |  | GAAGGTCGGAGTCAACGGATTTGATTAACAGCTCCCTGAAGAAC |  |
| IWB23706 | Excalibur_c21854_1154 | GAAGGTGACCAAGTTCATGCTGCAGACAGAAGGGTTTGCTTTA | TTGAGCTCGGGACTAGAGTG |
|  |  | GAAGGTCGGAGTCAACGGATTGCAGACAGAAGGGTTTGCTTTG |  |
| IWB52695 | Ra_c7974_1192 | GAAGGTGACCAAGTTCATGCTTCTTATATTATGGGACCGAGGGAA | GCCTTGTGCGTTTGAACATATATAA |
|  |  | GAAGGTCGGAGTCAACGGATTTCTTATATTATGGGACCGAGGGAG |  |
| IWA4188 | wsnp_Ex_c5356_9470772 | GAAGGTGACCAAGTTCATGCTGTTTTGGAGGCGAACTTTATACA | CCATTGCCATGTCTTTTAATCTCA |
|  |  | GAAGGTCGGAGTCAACGGATTGTTTTGGAGGCGAACTTTATACG |  |
| IWB51978 | Ra_c38371_1171 | GAAGGTGACCAAGTTCATGCTACCTACCAGTACCAAATCCCA | TGCTACGCGAGACTTCAGTG |
|  |  | GAAGGTCGGAGTCAACGGATTACCTACCAGTACCAAATCCCC |  |
| IWB50943 | Ra_c11468_305 | GAAGGTGACCAAGTTCATGCTGCTATCTCCTGCAAAAGAATGGTA | GCATTGTTCGATGGGCGTAT |
|  |  | GAAGGTCGGAGTCAACGGATTGCTATCTCCTGCAAAAGAATGGTG |  |
| IWB62994 | RAC875_rep_c73990_174 | GAAGGTGACCAAGTTCATGCTGCTTCTGTTTCAGGATTGGATGA | GACCTCTCCCTTCGCTGC |
|  |  | GAAGGTCGGAGTCAACGGATTGCTTCTGTTTCAGGATTGGATGG |  |
| IWB65103 | RFL_Contig5734_2005 | GAAGGTGACCAAGTTCATGCTTGAACTTCACTTGAGCGTACAT | CATCGCACGGCTAGCTCG |
|  |  | GAAGGTCGGAGTCAACGGATTTGAACTTCACTTGAGCGTACAG |  |
| *Stumpy* (qRT-PCR) | | CAGCATATGTTGAGGGTACC | CTAGGTGGTTCTAACAAGTC |
| *β−ACTIN* | | GACCGTATGAGCAAGGAGAT | CAATCGCTGGACCTGACTC |

*SNP names provided by Chinese Spring RefSeq v1.0 assembly (International Wheat Genome Sequencing Consortium 2018).

** KASP primers include the tail sequences for detection and the specific sequence for the SNP.

**Supplemental Table S2.** Monosaccharide linkage analysis of root cell walls comparing WT to *Stumpy* (backcrossed 4 times to WT) at permissive (0.5 mM) and non-permissive (10 mM) Ca^2+^ treatments.

| Monosaccharide* | Deduced linkage | WT  (%) | SD | *Stumpy*  (%) | SD | *P* |
| --- | --- | --- | --- | --- | --- | --- |
| **0.05 mM CaCl_2_** | | | | | | |
| **Ara (f)** | **terminal** | **8.55** | 0.53 | **8.70** | 0.34 | ns |
|  | **1,2-** | **0.66** | 0.07 | **0.77** | 0.02 | * |
|  | **1,3-** | **0.79** | 0.07 | **0.90** | 0.10 | ns |
|  | **1,5-** | **0.65** | 0.09 | **0.67** | 0.07 | ns |
| **Ara (p)** | **terminal** | **0.77** | 0.08 | **0.89** | 0.09 | * |
| **Xyl (p)** | **terminal** | **4.09** | 0.44 | **4.34** | 0.30 | ns |
|  | **1,2-** | **2.54** | 0.16 | **2.28** | 0.21 | ns |
|  | **1,3-** | **0.19** | 0.02 | **trace** |  | ns |
|  | **1,4-** | **13.94** | 1.40 | **12.91** | 1.20 | ns |
|  | **1,2,4-** | **0.89** | 0.15 | **0.95** | 0.17 | ns |
|  | **1,3,4-** | **5.53** | 0.48 | **5.33** | 0.43 | ns |
|  | **1,2,3,4-** | **0.84** | 0.40 | **0.75** | 0.06 | ns |
| **Man (p)** | **1,4-** | **0.56** | 0.09 | **0.59** | 0.08 | ns |
| **Gal (p)** | **terminal** | **2.44** | 0.17 | **2.77** | 0.22 | * |
|  | **1,6-** | **0.61** | 0.08 | **0.62** | 0.06 | ns |
|  | **1,3.6-** | **0.34** | 0.06 | **0.35** | 0.05 | ns |
| **Glc (p)** | **terminal** | **1.56** | 0.19 | **1.60** | 0.12 | ns |
|  | **1,4-** | **51.20** | 0.95 | **51.33** | 1.34 | ns |
|  | **1,4,6-** | **1.15** | 0.14 | **1.31** | 0.14 | ns |
| **GlcA (p)** | **terminal** | **1.56** | 0.19 | **1.60** | 0.12 | ns |
| **GalA (p)** | **1,4-** | **0.44** | 0.04 | **0.42** | 0.01 | ns |
| **DE** |  | **27.00** | 1.00 | **26.33** | 1.53 | ns |
|  |  |  |  |  |  |  |
| **10 mM CaCl_2_** | | | | | | |
| **Ara (f)** | **terminal** | **7.59** | 0.34 | **8.13** | 0.11 | ns |
|  | **1,2-** | **0.77** | 0.05 | **0.91** | 0.06 | * |
|  | **1,3-** | **0.81** | 0.02 | **1.16** | 0.09 | * |
|  | **1,5-** | **0.58** | 0.03 | **0.72** | 0.05 | * |
| **Ara (p)** | **terminal** | **0.75** | 0.02 | **0.96** | 0.08 | * |
| **Xyl (p)** | **terminal** | **3.32** | 0.08 | **4.11** | 0.14 | ** |
|  | **1,2-** | **2.79** | 0.02 | **2.21** | 0.17 | * |
|  | **1,3-** | **0.22** | 0.02 | **0.31** | 0.04 | * |
|  | **1,4-** | **15.80** | 0.14 | **12.53** | 0.95 | * |
|  | **1,2,4-** | **1.03** | 0.00 | **1.17** | 0.12 | ns |
|  | **1,3,4-** | **5.81** | 0.44 | **6.00** | 0.22 | ns |
|  | **1,2,3,4-** | **0.77** | 0.16 | **1.05** | 0.27 | ns |
| **Man (p)** | **1,4-** | **0.56** | 0.01 | **0.67** | 0.14 | ns |
| **Gal (p)** | **terminal** | **2.43** | 0.09 | **3.17** | 0.15 | ** |
|  | **1,6-** | **0.60** | 0.04 | **0.71** | 0.06 | ns |
|  | **1,3.6-** | **0.36** | 0.05 | **0.44** | 0.06 | ns |
| **Glc (p)** | **terminal** | **1.86** | 0.06 | **2.00** | 0.14 | ns |
|  | **1,4-** | **49.91** | 0.53 | **48.92** | 0.65 | ns |
|  | **1,4,6-** | **0.98** | 0.04 | **1.40** | 0.18 | * |
| **GlcA (p)** | **terminal** | **1.86** | 0.06 | **2.00** | 0.14 | ns |
| **GalA (p)** | **1,4-** | **0.43** | 0.01 | **0.52** | 0.02 | ** |
| **DE** |  | **27.67** | 1.53 | **26.67** | 2.08 | ns |

*See Pettolino et al. (2012) for a description of the monosaccharides and their linkages.

SD: standard deviation (n=3). *P* values for significant differences were calculated by Student’s *t*-test (ns not significant; * *P* < 0.05, ** *P* < 0.01).

DE: Degree of esterification on 1,4-GalA (p)

**Table S3.** Sequence analysis of a selection of mutants from the Cadenza TILLING population.

| Mutant ID | Mutation confirmed by sequencing? | Mutation in protein (missense or truncation) | Amino acid  residue* |
| --- | --- | --- | --- |
| Cadenza1719 | Yes | Val/Ilu | 830 |
| Cadenza1447 | Yes | Ala/Thr | 887 |
| Cadenza0882 | No |  |  |
| Cadenza0798 | Yes | Stop codon | 264 |
| Cadenza0313 | No |  |  |
| Cadenza 1339 | Yes | Arg/Gln | 700 |
| Cadenza1467 | Yes | Ala/Thr | 928 |
| Cadenza0124 | No |  |  |

*Position of amino acid residue or stop codon in predicted protein. The mutation in *Stumpy* substitutes Ala at position 113 with Val.

**Supplemental** **Text S1.**

**Conservation of root trichoblast volume concept.**

In *Stumpy,* the cell body of trichoblasts in roots shorten while the root hairs elongate under high Ca^2+^ conditions. The question is whether the total volume is the same? It appears to be close as these rough calculations suggest using data for cell sizes. At low Ca^2+^ the root cells (basal) of *Stumpy* root cells are about 360 μm long and at high Ca^2+^ these cells are about 66 μm long (Fig. 5B). At low Ca^2+^ the root hair cells of *Stumpy* root cells are about 110 μm long and at high Ca^2+^ they are about 1200 μm long (Fig. 3) and these cells are about two-fold longer than they are wide. The root cell diameters are ~30 μm (radius ~15 μm) and the root hair diameters in wheat are about 15 μm (radius 7.5 μm; (Hendriks et al. (2022)). Therefore, volumes (= π *r*^2^*l*) of *Stumpy* cells at low and high Ca^2+^ are:

*Stumpy* root trichoblast cell body volume

Low Ca^2+^ High Ca^2+^

V_b_ = π x 15 x 15 x 360 = 254,500 μm ^3^ V_b_ = π x 15 x 15 x 66 = 46,660 μm ^3^

*Stumpy* root hair volume

Low Ca^2+^ High Ca^2+^

V_h_ = π x 7.5 x 7.5 x 110 = 19,440 μm ^3.^  V_h_ = π x 7.5 x 7.5 x 1200 = 212,100 μm ^3^

Total cell volume (V_b +_ V_h_ )

Low Ca^2+^ High Ca^2+^

V_b +_ V_h_ = (254,500 + 19,440) V_b +_ V_h_ = (46,660 + 212,100)

≈ 273,940 μm ^3^ ≈ 258,750 μm ^3^

In summary, total cell volumes are about 260,000 μm ^3^ at low Ca^2+^ compared with 247,000 μm ^3^ at high Ca^2+^ which are ~ 6% different from one another, and therefore very similar for these approximate calculations.

**Hendriks PW, Ryan PR, Hands P, Rolland V, Gurusinghe S, Weston LA, Rebetzke GJ, Delhaize E** (2022) Selection for early shoot vigour in wheat increases root hair length but reduces epidermal cell size of roots and leaves. J Exp Bot **73:** 2499-2510


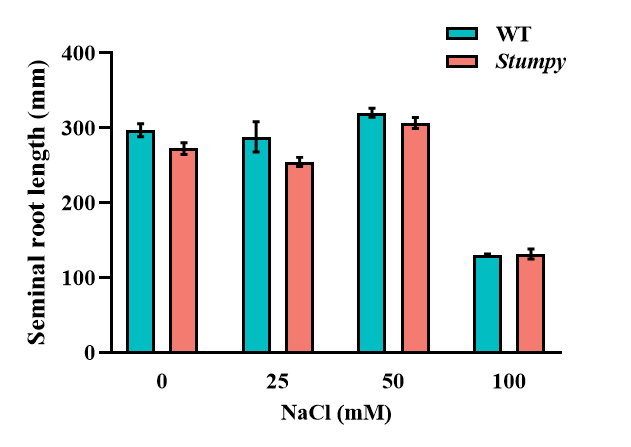


**Figure S1.** High ionic strength does not control the *Stumpy* phenotype. Seedlings of WT and *Stumpy* (backcrossed 4 times to WT) were grown for 3 days in nutrient solution supplemented with a range of NaCl concentrations. Error bars denote the SE and no significant differences between genotypes were identified for any of the treatments as determined with Student’s *t*-test (n = 6).


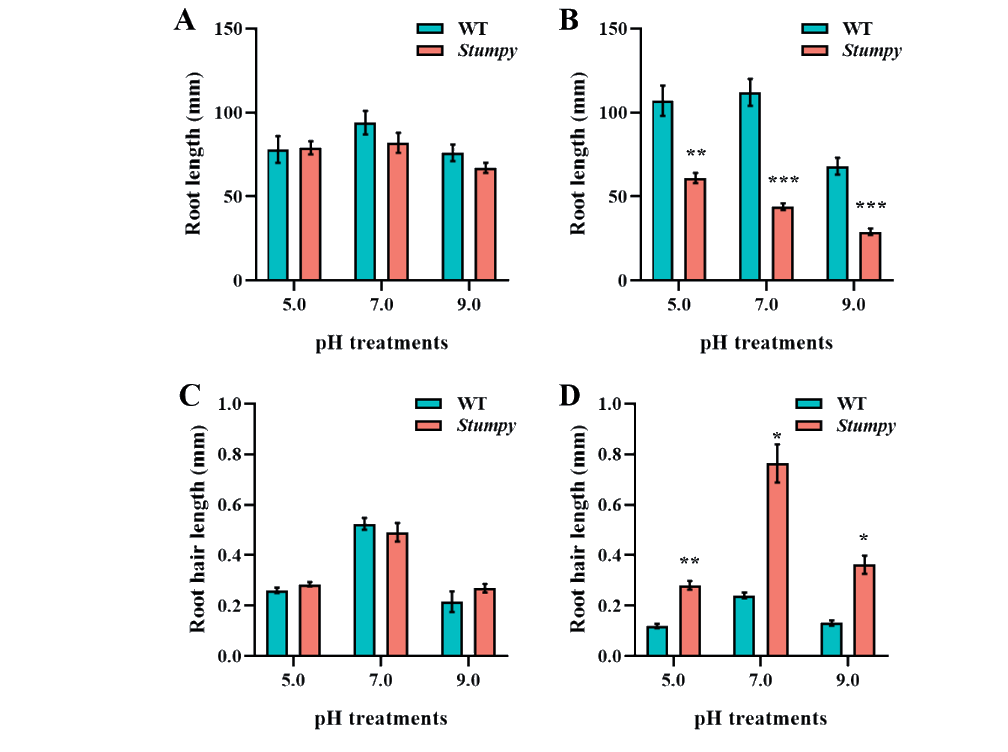


**Figure S2.** Effect of pH on appearance of the *Stumpy* phenotype. Seedlings of WT and *Stumpy* (backcrossed 4 times to WT) were grown for 4 days in nutrient solutions at pH values of 5, 7 and 9 supplemented with 0.5 mM (**A** and **C**) or 10 mM CaCl_2_ (**B** and **D**). Root lengths (**A** and **B**) and root hair lengths (**C** and **D**) were measured at the end of the experiment. Error bars denote the SE and data comparing WT to *Stumpy* at each pH treatment was analysed with Student’s *t*-test to identify significant differences between genotypes (n = 6; * *P* < 0.05; ** *P* < 0.01; *** *P* < 0.001)).


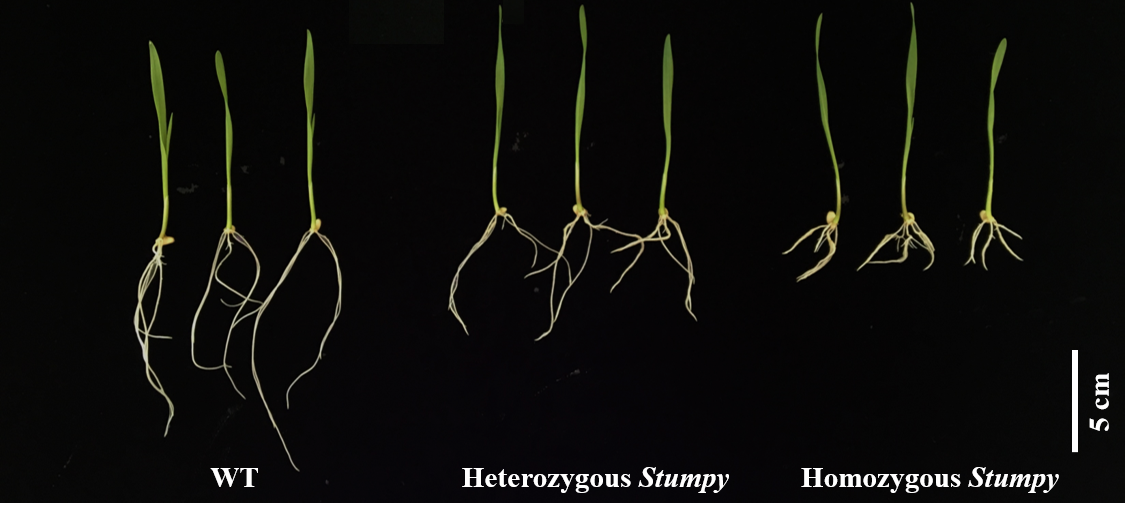


**Figure S3.** Phenotypic classes of *Stumpy* plants in segregating F_2_ seedlings suggest *Stumpy* is a semi-dominant mutation. Since the phenotypes of heterozygous and homozygous *Stumpy* seedlings sometimes overlapped, data for both classes were combined as mutant-like phenotypes when calculating segregation ratios (bar indicates 5 cm).


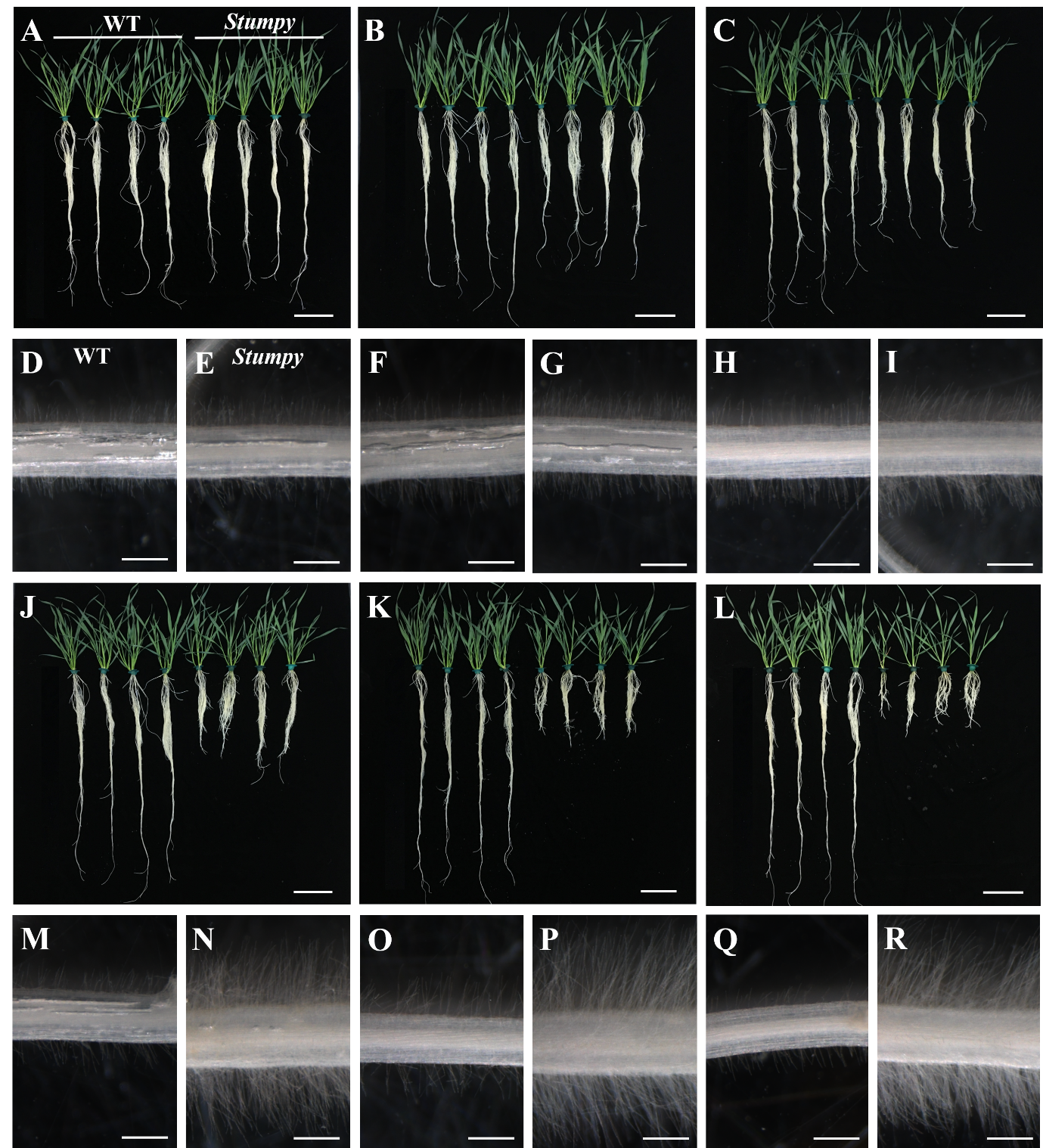


**Figure S4.** The *Stumpy* phenotype under various external Ca^2+^ concentration. Whole plant (bar indicates 10 cm) and root hair (bar indicates 1 mm) phenotypes of WT and *Stumpy* (backcrossed 4 times to WT) plants grown by hydroponics in various nutrient solution supplemented with 0.5 mM (A, D, E), 1 mM (B, F, G), 2 mM (C, H, I), 4 mM (J, M, N), 8 mM (K, O, P), 10 mM (L, Q, R) CaCl_2_.

**
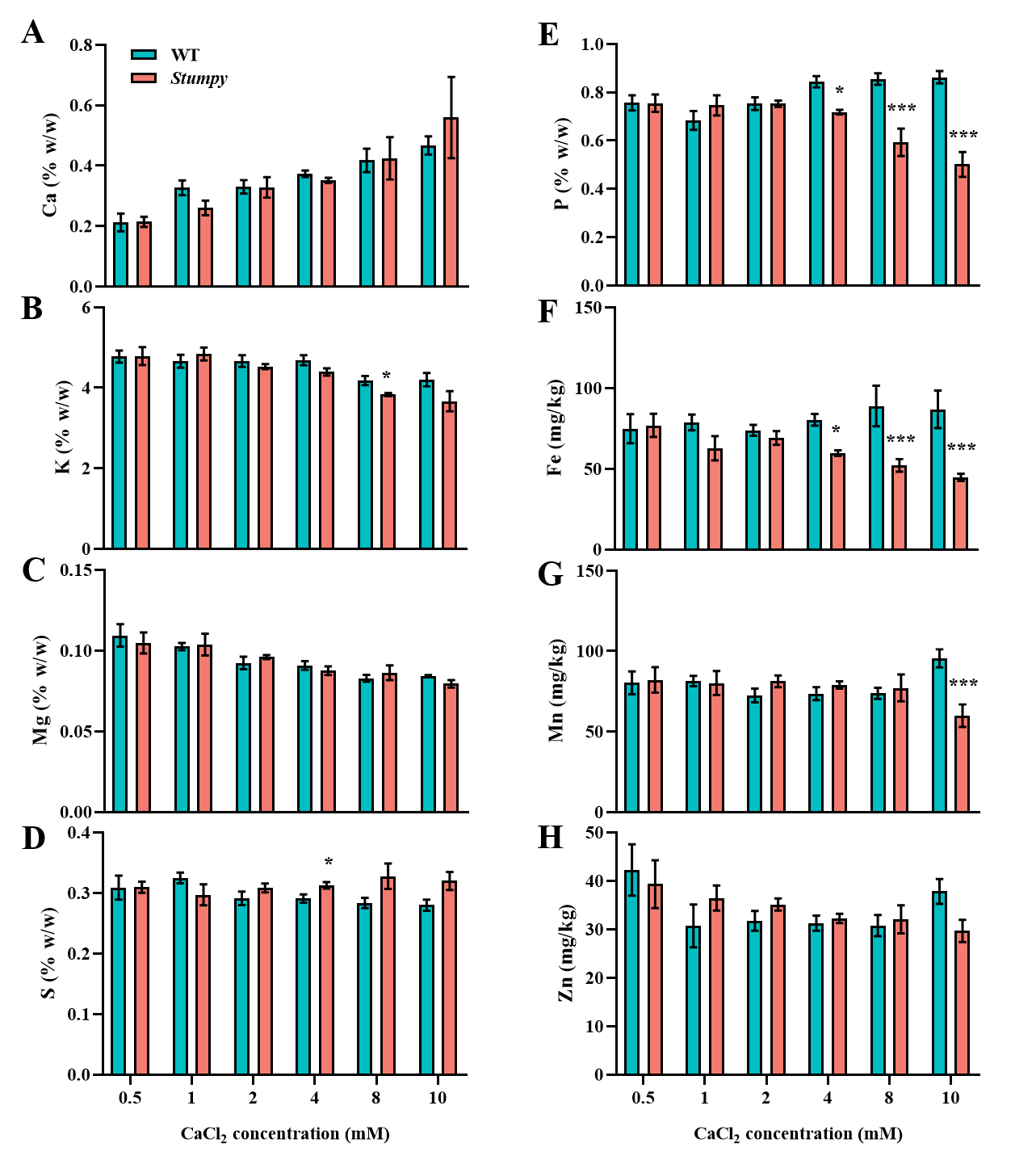
**

**Figure S5.** Elemental concentrations in shoots of WT and *Stumpy*. **A)** calcium, **B)** potassium, **C)** magnesium, **D)** sulfur, **E)** phosphorus, **F)** iron, **G)** manganese and **H)** zinc concentrations in shoots of WT and *Stumpy* (backcrossed 4 times to WT). Plants were grown for 20 days in hydroponics. Values are the mean ± SE (n=4). Within each panel, columns marked with asterisks indicate significantly different means between genotypes at each CaCl_2_ treatment as determined by Student’s *t*-test (* *P* < 0.05; ** *P* < 0.01; *** *P* < 0.001).


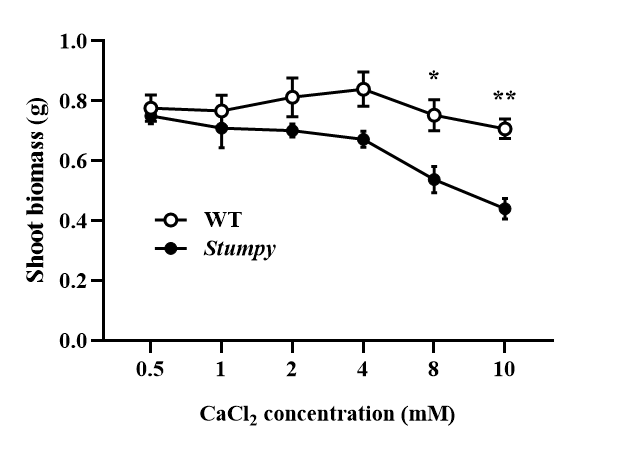


**Figure S6.** Shoot biomass of WT (empty symbols) and *Stumpy* (filled symbols) grown for 20 days by hydroponics supplemented with CaCl_2_ concentrations ranging from 0.5 to 10 mM. The *Stumpy* line was backcrossed four times to WT. Error bars on symbols indicate SE (n=6) and asterisks indicate statistical significance between genotypes at the CaCl_2_ concentrations as determined by Student’s *t*-test (**P* < 0.05; ** *P*<0.01).


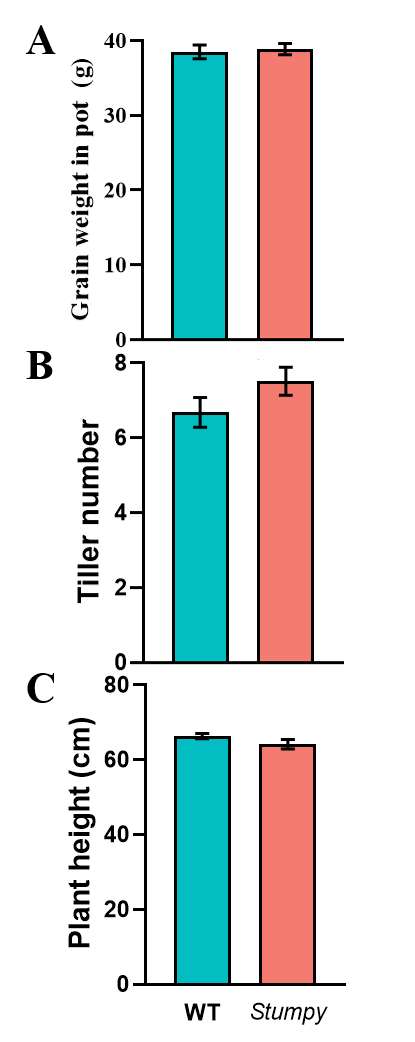


**Figure S7.** *Stumpy* has a similar phenotype to WT when grown under permissive conditions. Plants were grown to maturity in a glasshouse on potting mix not amended with lime. At harvest, **A)** grain weight **B)** tiller number and **C)** plant height were recorded for WT and *Stumpy* that had being backcrossed twice to WT. Error bars indicate the SE (n=4) and a Student’s *t*-test did not identify significant differences between WT and *Stumpy* for any of the parameters.


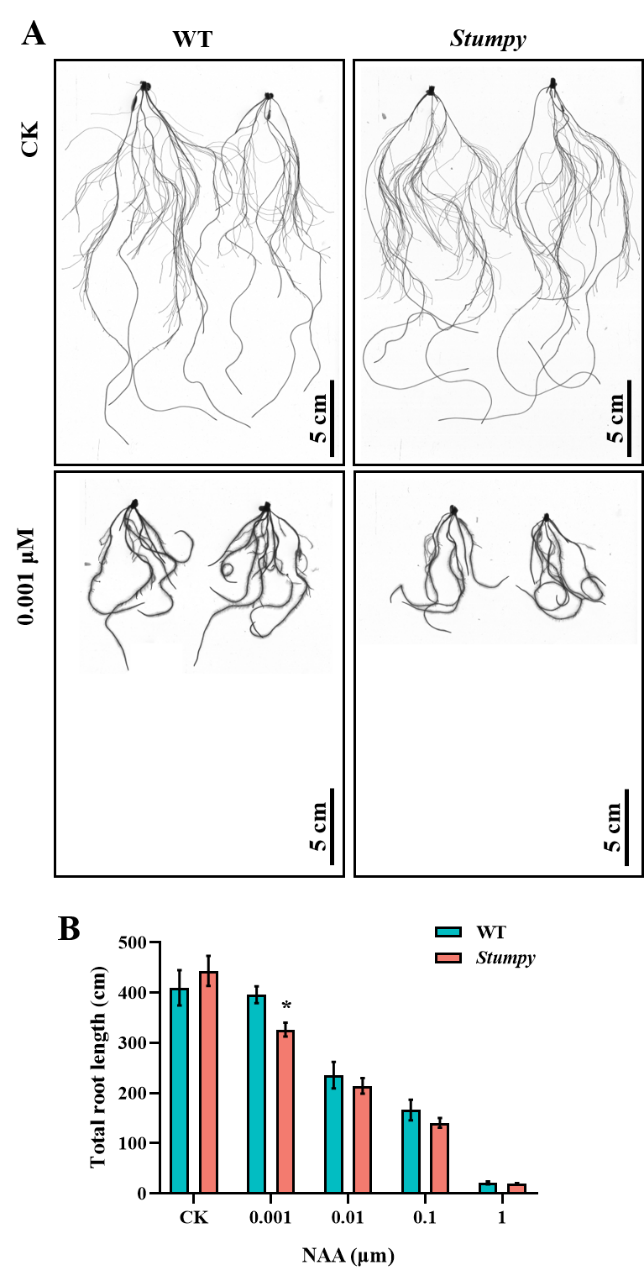

**Supplemental Figure S8.** WT and *Stumpy* (backcrossed 4 times to WT) root responses to auxin and effect of Ca^2+^ on internal auxin concentration. **A)** Wheat seedlings grown with high concentrations of external auxin superficially phenocopy roots of the *Stumpy* mutant grown with high Ca^2+^. WT seedlings were grown in hydroponic nutrient solution with a low Ca^2+^ concentration in absence of added auxin (top panels) or in the same nutrient solution with the addition of 0.1 mM of the auxin analog 1-naphthalene acetic acid (NAA, bottom panels; CK is the check control solution without added NAA). **B)** WT and *Stumpy* showed a similar dose response to NAA for root elongation when grown in hydroponic culture. *Stumpy* was marginally more sensitive of NAA at 0.001 mM NAA but otherwise did not differ from WT (Student’s *t*-test used to compare genotypes at each NAA concentration; * *P* < 0.05; n = 6). Internal IAA concentrations of whole roots **C)** and shoots **D)** of WT and *Stumpy* (mutant) at permissive (0.5 mM) and non-permissive (10 mM) Ca^2+^ concentrations. Bars with different letters indicate significant differences at P < 0.05 as determined with a 2-way ANOVA (n = 5).


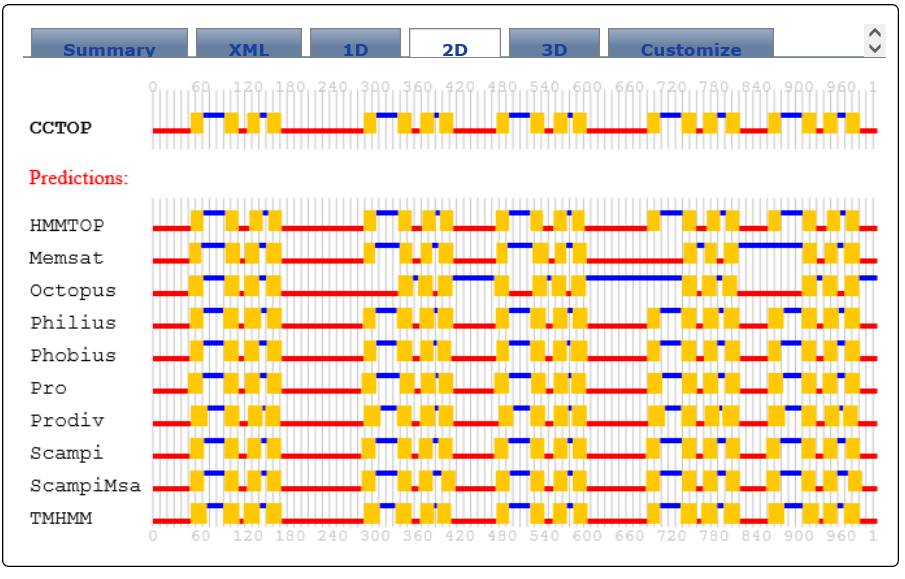
**Figure S9.** *TraesCS7B03G0323100* encodes a membrane protein. The amino acid sequence was submitted to CCTOP (<https://cctop.ttk.hu>) which predicts topology based on a consensus of 10 different methods. The CCTOP prediction is at the top of the various schematics with predictions from the various methods shown underneath. The dark yellow boxes are predicted to be transmembrane domains while blue indicates regions predicted to be outside the cell and red indicates regions predicted to be inside the cell. The mutation in *Stumpy* changes an alanine at position 113 to valine in the second predicted transmembrane domain.


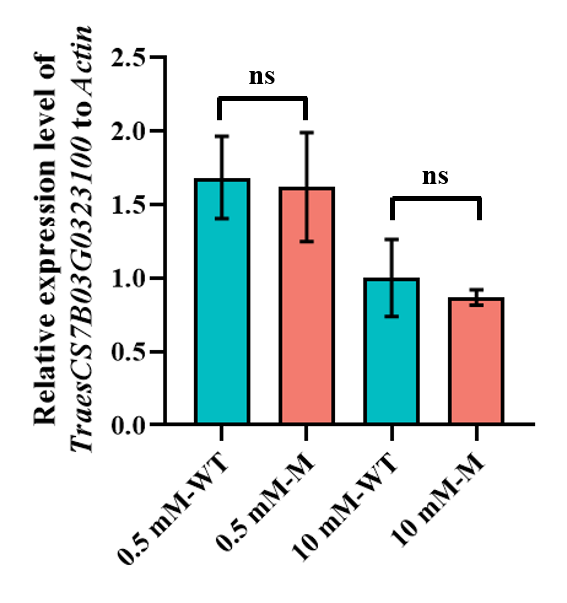


**Figure S10.** *TraesCS7B03G0323100* is expressed to a similar level in roots of WT (turquoise columns) and *Stumpy* (magenta columns; M, backcrossed four time to WT) at both permissive (0. 5 mM) and non-permissive (10 mM) Ca^2+^ concentrations. *TraesCS7B03G0323100* expression level was assessed by qRT-PCR and is expressed relative to expression of the actin gene. Error bars indicate the SE and Student’s *t*-test (n = 3) did not identify significant differences between WT and *Stumpy* for either treatment.


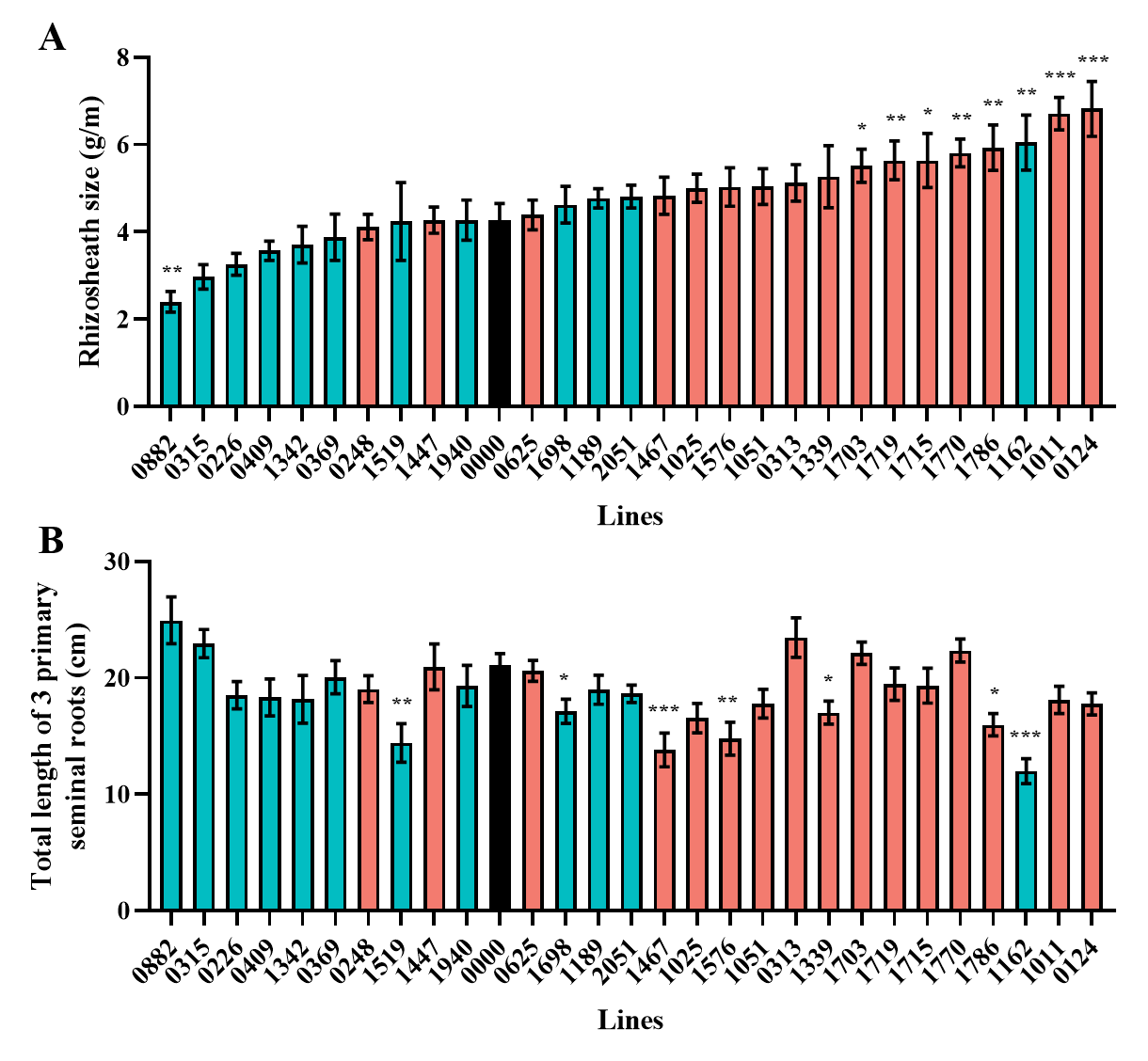


**Figure S11.** Screening of the Cadenza TILLING population with mutations in *TraesCS7B03G0323100* for rhizosheath size and root length. The Cadenza TILLING population was searched on-line for lines that had a mutation within the *TraesCS7B03G0323100* gene. Seedlings were screened for **A)** rhizosheath size and **B)** length of the three primary seminal roots in a soil obtained from the Dundee experimental station that had been amended with 30 g CaCO_3_/ kg soil. For A) the lines were ordered from those with the smallest rhizosheath to the largest and for B) the same order was maintained for root length showing no relationship between the two parameters. The numbers on the x-axis indicate the specific Cadenza lines with most predicted to have non-synonymous mutations. Bars in magenta are lines that were found to have large rhizosheaths when screened visually on limed Robertson soil in Canberra. Cadenza0882 was included as it had a predicted stop codon within the coding region although sequencing did not identify the mutation within the seed batch (see **Table S3**). The the black bar indicates the control WT line Cadenza0000. The error bars denote the SE (n = 6) and asterisks denote significant differences of lines compared to Cadenza0000 (uncorrected Fischer’s LSD: * P < 0.05; ** P < 0.01; *** P < 0.001).


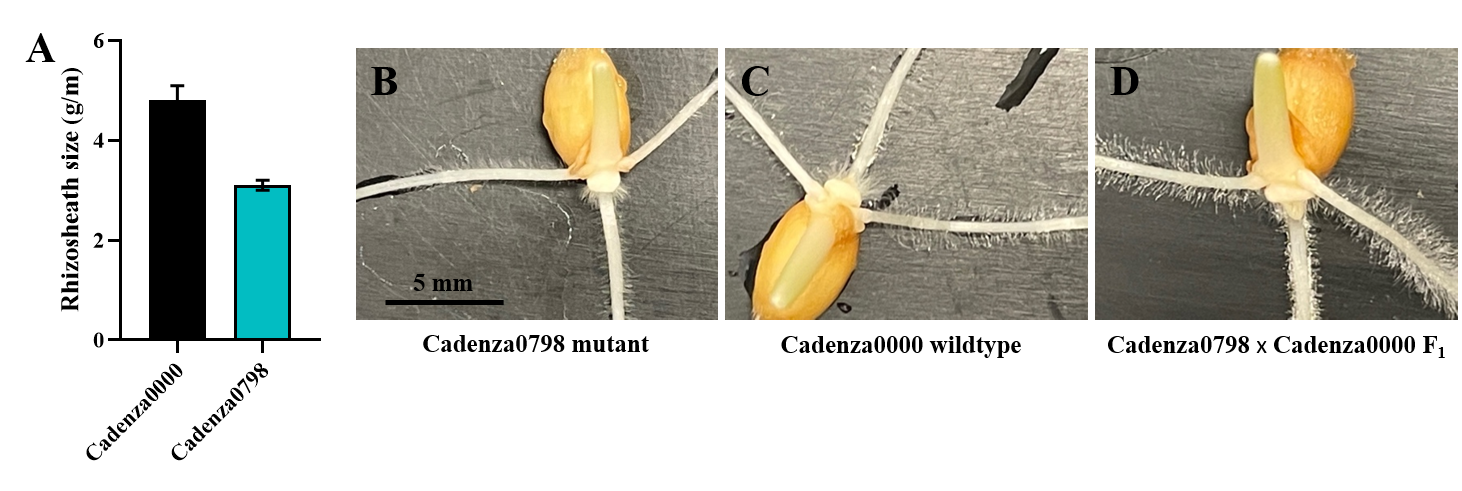


**

**Figure S12.** Cadenza0798 with a stop codon in *TraesCS7B03G0323100* has a smaller rhizosheath than WT Cadenza0000 and is a recessive root hair mutant. **A)** Rhizosheath sizes of Cadenza0000 and Cadenza0798. Root hair phenotypes of seedlings grown on Petri dishes of **B)** Cadenza0798 **C)** Cadenza 0000 and **D)** an F_1_ resulting from a cross between the two parental genotypes.


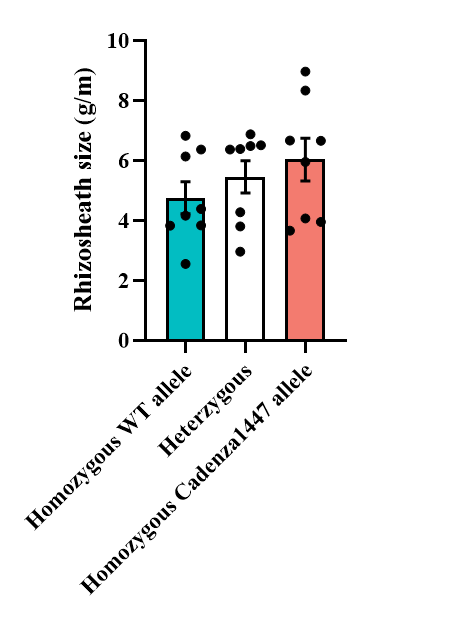


**Figure S13.** Rhizosheath sizes of F_2_ seedlings generated from a cross between Cadenza0000 (WT) and mutant Cadenza1447 (large rhizosheath). Seedlings were analysed with a KASP marker to determine which allele of gene *TraesCS7B03G0323100* they possessed. Columns show mean rhizosheath sizes of 8 seedlings that were homozygous WT, homozygous Cadenza1447 or heterozygous for *TraesCS7B03G0323100* alleles. Error bars denote the SE and although mean values are consistent with allele types, individual seedlings do not show a clear co-segregation.
